# Serotype-specific epidemiological patterns of inapparent versus symptomatic primary dengue virus infections: a 17-year cohort study in Nicaragua

**DOI:** 10.1016/S1473-3099(24)00566-8

**Published:** 2025-03

**Authors:** Sandra Bos, José Victor Zambrana, Elias Duarte, Aaron L Graber, Julia Huffaker, Carlos Montenegro, Lakshmanane Premkumar, Aubree Gordon, Guillermina Kuan, Angel Balmaseda, Eva Harris

**Affiliations:** a Division of Infectious Diseases and Vaccinology, School of Public Health, University of California, Berkeley, Berkeley, CA, USA; b Sustainable Sciences Institute, Managua, Nicaragua; c Department of Epidemiology, School of Public Health, University of Michigan, Ann Arbor, MI, USA; d Department of Microbiology and Immunology, University of North Carolina School of Medicine, Chapel Hill, NC, USA; e Centro de Salud Sócrates Flores Vivas, Ministerio de Salud, Managua, Nicaragua; f Laboratorio Nacional de Virología, Centro Nacional de Diagnóstico y Referencia, Ministerio de Salud, Managua, Nicaragua

## Abstract

**Background:**

Dengue is the most prevalent mosquito-borne viral disease and a major public health problem worldwide. Most primary infections with the four dengue virus serotypes (DENV1–4) are inapparent; nonetheless, whether the distribution of symptomatic versus inapparent infections by serotype varies remains unknown. Here, we present (1) the evaluation of a DENV1–4 envelope domain III multiplex microsphere-based assay (EDIII-MMBA) to serotype inapparent primary infections and (2) its application leveraging 17 years of prospective sample collection from the Nicaraguan Pediatric Dengue Cohort Study (PDCS).

**Methods:**

We analysed primary DENV infections in the PDCS from 2004 to 2022 detected by inhibition ELISA (iELISA) or RT-PCR. First, we evaluated the performance of the EDIII-MMBA for serotyping with samples characterised by RT-PCR or focus reduction neutralisation test. Next, we analysed a subset of inapparent primary DENV infections in the PDCS with the EDIII-MMBA to evaluate the epidemiology of inapparent infections. Remaining infections were inferred using stochastic imputation, taking year and neighbourhood into account. Infection incidence and percentage of inapparent, symptomatic, and severe infections were analysed by serotype.

**Findings:**

Between Aug 30, 2004, and March 10, 2022, a total of 5931 DENV-naive participants were followed in the PDCS. There were 1626 primary infections (382 symptomatic, 1244 inapparent) detected by iELISA or RT-PCR over the study period. The EDIII-MMBA demonstrated excellent overall accuracy (100%, 95% CI 95·8–100) for serotyping inapparent primary DENV infections when evaluated against gold-standard serotyping methods. Of the 1244 inapparent infections, we analysed 574 (46%) using the EDIII-MMBA. We found that the majority of primary infections were inapparent, with DENV3 exhibiting the highest likelihood of symptomatic (pooled odds ratio compared with DENV1: 2·13, 95% CI 1·28–3·56) and severe (6·75, 2·01–22·62) primary infections, whereas DENV2 was similar to DENV1 in both analyses. Considerable within-year and between-year variation in serotype distribution between symptomatic and inapparent infections and circulation of serotypes undetected in symptomatic cases were observed in multiple years.

**Interpretation:**

Our study indicates that case surveillance skews the perceived epidemiological footprint of DENV. We reveal a more complex and intricate pattern of serotype distribution in inapparent infections. The substantial differences in infection outcomes by serotype emphasises the need for vaccines with balanced immunogenicity and efficacy across serotypes.

**Funding:**

National Institute of Allergy and Infectious Diseases (National Institutes of Health) and Bill & Melinda Gates Foundation.

**Translation:**

For the Spanish translation of the abstract see Supplementary Materials section.

## Introduction

Dengue, caused by the four dengue virus serotypes (DENV1–4), is the most prevalent mosquito-borne viral disease in humans and a major global health threat.^1^ Infection outcomes range from inapparent or subclinical infection to severe, potentially fatal disease. The global dissemination of DENV, alongside the co-circulation of its serotypes, has increased both the incidence and severity of the disease in recent decades.^1^, ^2^ This epidemiological shift emphasises the need for a comprehensive understanding of the circulation and interactions among the different serotypes and their implications for disease outcomes.

Infection with one of the four antigenically related DENV serotypes leads to the production of type-specific antibodies as well as cross-reactive antibodies that can bind to other serotypes. These antibodies can either protect against or increase the risk of subsequent symptomatic or severe DENV infection in both natural infections and vaccines.^3^, ^4^, ^5^ In this context, one factor that can influence clinical outcome is the infecting serotype.^5^, ^6^ Furthermore, an association between the order of DENV serotype infection and clinical outcome has been reported,^6^ suggesting that the initial infecting serotype can modulate infection outcomes.^5^, ^7^, ^8^

**Research in context**

**Evidence before this study**

We conducted a search in PubMed for studies published from inception to Feb 4, 2024. Keywords included “dengue virus” and “DENV” in combination with “inapparent infections”, “asymptomatic infections”, “primary infections by serotype”, “FoI by serotype”, “force of infection”, and “force of infection by serotype”. Our search identified a substantial gap in the current understanding of dengue epidemiology. Despite acknowledging the high prevalence of inapparent dengue virus (DENV) infections in endemic regions, previous research has focused primarily on symptomatic infections, potentially biasing our understanding of the DENV epidemiological landscape and hindering our capacity to determine the complete disease spectrum of the different DENV serotypes. While cross-sectional studies have provided preliminary insights into this gap, there is a need for more comprehensive and detailed serotype-specific insights to evaluate the epidemiological impact of inapparent infections. The lack of comprehensive characterisation of inapparent infections reflects methodological challenges, particularly the need for prospective cohort studies designed to capture and accurately serotype these infections. Moreover, the reliance on labour-intensive and low-throughput antibody neutralisation assays for serotyping, despite their accuracy, has constrained high-throughput analysis required for large-scale epidemiological studies.

**Added value of this study**

Our study, spanning 17 years of prospective cohort data in Nicaragua, addresses this bottleneck in dengue research by providing a detailed examination of primary inapparent infections. The introduction of a novel envelope domain III multiplex microsphere-based assay for DENV serotyping represents a major methodological advance, offering an efficient, scalable alternative for large epidemiological studies. A key contribution of our study is the intricate pattern of serotype distribution among inapparent infections. In contrast to the serotype predominance observed in symptomatic infections, inapparent infections exhibit a complex landscape with co-circulation of multiple DENV serotypes, including serotypes undetected in symptomatic surveillance in multiple years. Our systematic documentation of the entire disease spectrum provides unprecedented insights into the serotype-specific disease burden in primary infection, including the proportion of symptomatic versus inapparent infection and its temporal variations, thus providing a more complete picture of DENV epidemiology than has been available to date. Notably, we demonstrate striking differences in disease severity by serotype, with DENV3 infections being considerably more symptomatic and more severe compared with DENV1 and DENV2, the latter displaying the highest rate of inapparent primary infection.

**Implications of all the available evidence**

Our research challenges previous assumptions by demonstrating that inapparent and symptomatic primary DENV infections present distinct epidemiological profiles, revealing that the epidemiological footprint of DENV is broader and more nuanced than previously recognised through symptomatic cases alone. These findings underscore the utility of continuous and comprehensive surveillance systems that capture both symptomatic and inapparent infections to accurately assess the epidemiological burden of DENV and inform public health interventions. Additionally, they provide critical insight for enhancing the accuracy of predictive DENV transmission modelling. Furthermore, the marked differences in infection outcomes by serotype emphasise the need for serotype-informed public health strategies. This nuanced understanding is pivotal for the crafting of targeted interventions, vaccine development and vaccination strategies, and efficient resource allocation, ultimately contributing to the global effort to mitigate the impact of dengue.

While the majority of primary DENV infections are inapparent, they contribute substantially to the force of infection, transmission, and overall epidemiological burden.^9^, ^10^ However, accurately assessing this burden, understanding the complete disease spectrum associated with each serotype, and predicting the risk of severe disease outcomes upon subsequent infection have been challenging due to the lack of characterisation of inapparent infections. This knowledge gap stems from methodological limitations, particularly the need for prospective cohort studies designed to capture and serotype inapparent infection, compounded by the low-throughput nature of neutralisation assays that are used to identify serotype in inapparent infections. Consequently, previous research has predominantly focused on symptomatic infections, potentially skewing our understanding of the DENV epidemiological landscape.

To address this bottleneck in dengue research, we leveraged 17 years of prospective sample collection from participants enrolled in our paediatric cohort in Nicaragua and demonstrated the hidden contribution of inapparent infection to both the overall and serotype-specific DENV burden in Nicaragua between 2004 and 2022. To achieve this, we used an envelope domain III multiplex microsphere-based assay (EDIII-MMBA), previously validated to differentiate DENV and Zika virus (ZIKV) infections and determine the number of prior DENV infections, to serotype inapparent DENV infection.^11^ We then examined the distribution of DENV serotypes in inapparent versus symptomatic primary infections, the proportion of inapparent infections per serotype, and their temporal dynamics in the paediatric cohort, together revealing the full disease spectrum associated with primary DENV infection by serotype.

## Methods

### Study design and participants

In this study, we first evaluated the performance of the EDIII-MMBA to serotype primary DENV infections and subsequently used it to characterise the serotype of inapparent primary infections and evaluate the epidemiological and clinical landscape of primary DENV infections in Nicaragua. To do so, we leveraged serum samples collected from 2004 to 2022 from our ongoing Pediatric Dengue Cohort Study (PDCS),^12^ which has followed around 4000 active participants annually aged 2–17 years since 2004. The PDCS is conducted at the Sócrates Flores Vivas Health Center located in District 2 of Managua, Nicaragua. Dengue cases are detected by enhanced passive surveillance, and healthy samples are collected annually in March–April, which allows the detection of inapparent infections. The last cohort year used for the present study was cohort year 2021, which ended in March, 2022. Ethics statements, description of the PDCS and participants, case definitions, and severity classifications are described in appendix 2 (pp 2–3).^12^ We included DENV-naive participants at enrolment and followed them until they experienced a primary DENV infection. Participants with DENV or ZIKV immunity at enrolment were excluded.

### Procedures

A primary DENV infection was defined among individuals who entered the cohort DENV-naive and ZIKV-naive, as measured by DENV inhibition ELISA (iELISA) and ZIKV NS1 blockade-of-binding ELISA, and who seroconverted to DENV. Symptomatic infections were confirmed by detection of DENV RNA by RT-PCR or virus isolation in the acute phase sample or seroconversion detected in paired acute-phase and convalescent-phase samples by DENV IgM capture ELISA or iELISA. Inapparent primary infections were detected by seroconversion between two consecutive annual samples by iELISA, in the absence of any documented symptomatic DENV infection, according to the case definition, in the intervening year. Disease severity of symptomatic infections was classified according to the 2009 WHO dengue guidelines. Procedures, case definitions, and assays are explained in detail in appendix 2 (pp 2–3).

The gold standard to serotype inapparent infections is the DENV1–4 neutralisation assay, which is both time-intensive and sample-intensive. To address these limitations, we evaluated the performance of a multiplex assay containing EDIII of DENV1–4 and ZIKV conjugated to avidin-coated microspheres via an Avi-Tag,^11^ the EDIII-MMBA, to serotype primary infections. The EDIII-MMBA was evaluated against RT-PCR and focus reduction neutralisation test (FRNT) using the evaluation set, which was made up of late convalescent samples from the annual sample collection (3–9 months post-infection) selected by convenience sampling (see appendix 2 p 3 for details). Of the inapparent primary infections that occurred from 2004 to 2022, a subset were processed by the EDIII-MMBA assay to study the epidemiology of primary DENV infection in the PDCS. Selection of this subset was performed by random selection of 40% of primary DENV-infected individuals each year, or 100% if fewer than 20 infections were recorded, restricted to those with serum or plasma available from the following annual sample collection (3–9 months post-infection). To improve precision, we also included all inapparent infection samples from the evaluation set.

### Outcomes

Outcomes included inapparent, symptomatic, severe, and overall primary DENV infections as defined in the Procedures section and appendix 2 (pp 2–3). Our primary endpoints included the ratio of symptomatic versus inapparent and severe versus non-severe infections by serotype. Our secondary analyses include a year-by-year analysis of the relative distribution of DENV serotypes in inapparent versus symptomatic infections, the percentage of symptomatic DENV infections among total primary infections by serotype over time, and the overall infection incidence by serotype over time.

### Statistical analysis

The accuracy of the EDIII-MMBA assay to serotype primary DENV infections was evaluated by comparing it with gold standards: RT-PCR (symptomatic) and FRNT (inapparent). Sensitivity, specificity, and accuracy, calculated as the sum of true positives and true negatives divided by the total number of samples evaluated, were calculated for each serotype (DENV1–4) and ZIKV across all test combinations. We calculated 95% CIs using the exact binomial method for all performance measures.

To determine population-level infection parameters over time by DENV serotype, including percentage of individuals infected among the DENV-naive population and percentage of severe, symptomatic, and inapparent primary infections among total primary infections, we addressed missingness of serotype in both inapparent and symptomatic primary infections by employing multiple imputation with stochastic multivariate imputation by chained equations (MICE). A polytomous logistic regression model, incorporating epidemic year and neighbourhood as predictors, was used to impute missing serotypes. We ran independent imputation models for inapparent and symptomatic infections, running 1000 simulations with ten iterations for both models. The resulting imputed datasets were merged for analysis, pooling all results across simulations. To understand temporal trends of infected individuals by outcome (symptomatic *vs* inapparent) for each serotype, we plotted the pooled counts and percentages across imputations over time. Additionally, we calculated the relative distribution of serotypes by outcomes over time.

For our primary endpoints, we analysed the ratio of symptomatic and severe infections among total infections specific to each serotype using separate logistic regression models by symptomatic and severe infections, adjusting by serotype and controlling by epidemic year. As secondary endpoints, we analysed within-year relative distribution of serotypes by inapparent and symptomatic infections by applying Fisher's exact test to both serotyped and imputed data. Additionally, we analysed changes in percentage of symptomatic DENV infections by serotype over time by applying logistic regression models measuring the likelihood of symptomatic infection by epidemic year and stratifying by serotype. Finally, we analysed the infection incidence proportion by serotype, applying intercept-only logistic regression, and by year, measuring the likelihood of infection. Since naive (susceptible) individuals can be counted more than once and are used in the denominator for incidence calculation, we also ran these models using a mixed model regression approach by maximum likelihood with an unstructured covariance matrix, using individual ID as the cluster term. A conceptual framework of our assumptions about the relationship between variables used in our analyses is shown in appendix 2 (p 6). A validation of our final imputation model and various sensitivity analyses on primary endpoints were performed (appendix 2 pp 6–7). To account for imputed data, we pooled analyses across simulations using Rubin's rule to obtain estimates and 95% CIs for model coefficients. All statistical analyses and data imputation were executed in R (version 4.3.2), using the “mice” package (version 3.16.0).

### Role of the funding source

The funders of the study had no role in study design, data collection, data analysis, data interpretation, or writing of the report.

## Results

**Figure 1:**
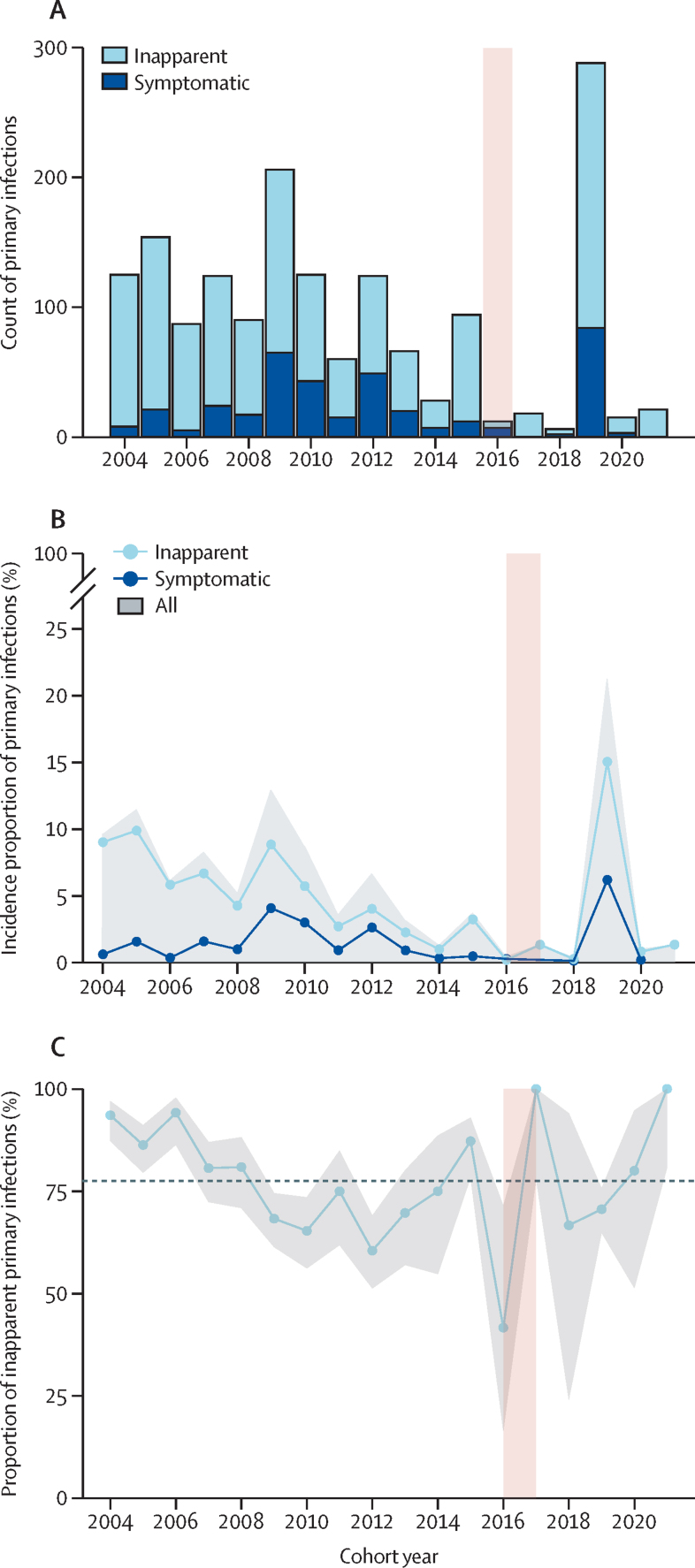
Overall symptomatic and inapparent DENV infections in the PDCS from 2004 to 2022 (A) Yearly counts of DENV primary infections by symptomatic or inapparent status, as indicated. (B) Yearly cumulative incidence proportion of primary inapparent and symptomatic DENV infections. (C) Yearly proportion of inapparent infections among total primary DENV infections. The pink shaded area in 2016 is the period when ZIKV circulated in the PDCS. The grey area in panel B covers the pooled incidence proportions of overall primary DENV infections by serotype. The dashed line in panel C is the yearly average of inapparent primary DENV infections across years, and the grey area represents the 95% CI. The x-axes show the cohort year; cohort year 2021 ended in March, 2022. DENV=dengue virus. PDCS=Pediatric Dengue Cohort Study. ZIKV=Zika virus.

Between Aug 30, 2004, and March 10, 2022, a total of 5931 DENV-naive participants were followed (median 1536 [IQR 1425–1861] per year), of whom 1626 experienced a primary infection as detected by RT-PCR or iELISA (appendix 2 p 8). 5824 participants with DENV or ZIKV seropositivity at enrolment were excluded. Notably, this period includes the ZIKV epidemic in 2016, which markedly dampened DENV circulation in Nicaragua in 2016–18. We first evaluated the count and incidence proportion of primary infections (figure 1A, B). Over the 17-year period, we observed considerable year-to-year fluctuations in the number of primary infections, ranging from five in 2018 to 287 in 2019, with a median annual incidence proportion of 4% (IQR 1–9; appendix 2 p 8). Before the emergence of ZIKV, a median of 105 (IQR 72–124) primary infections were recorded annually, which dropped to nine (7–12) from 2016 to 2018, before a major resurgence of DENV primary infections in 2019 (n=287). Out of the 1626 total primary infections we studied, 382 were symptomatic, while 1244 were inapparent. We found that a median of 77% (IQR 68–86) of primary infections was inapparent, although this percentage varied significantly across epidemic years (figure 1C).

**Figure 2:**
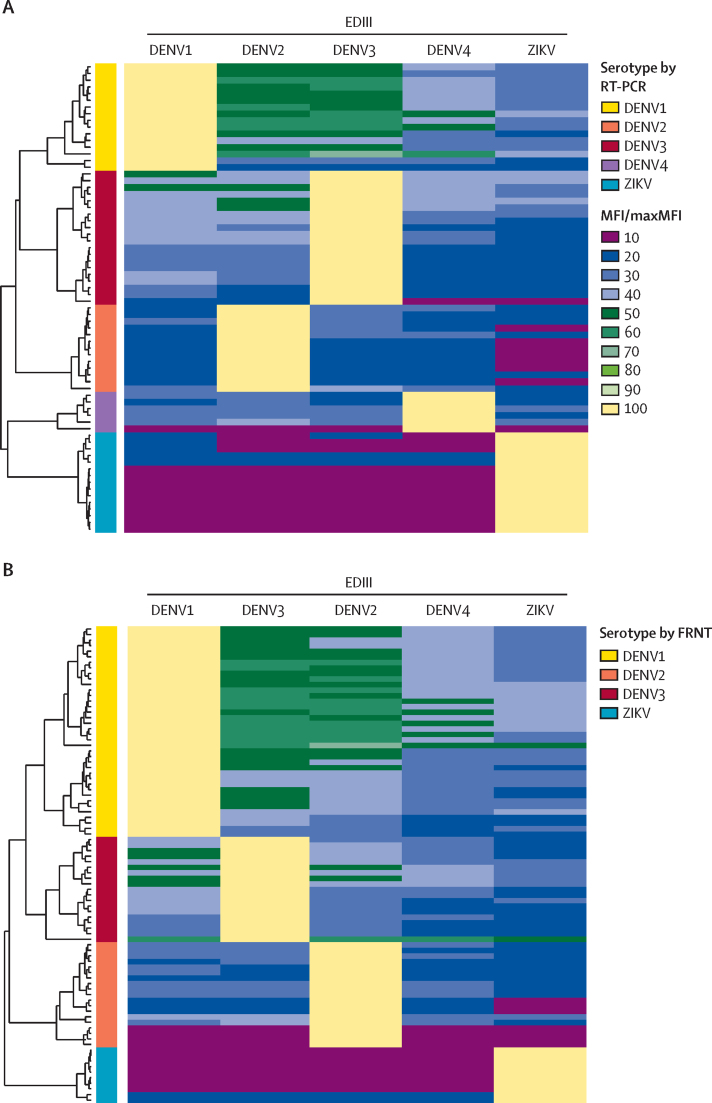
Concordance of relative EDIII-MMBA values from participants with primary DENV infection with values from RT-PCR and FRNT (A) Symptomatic primary DENV infections confirmed by RT-PCR. (B) Inapparent primary DENV infections confirmed by FRNT. Within the heatmaps, each cell indicates the MFI. MFI values per participant were transformed by dividing the MFI of each EDIII antigen by the highest MFI observed in the set (MFI/maxMFI). Adjacent to each heatmap, dendrograms display the hierarchical clustering of individuals based on Euclidean distances calculated for the normalised MFI values. On the right of the dendrograms, a colour-coded column classifies individuals according to the gold standard assay results, providing a reference for true infection status. DENV=dengue virus. EDIII-MMBA=envelope domain III multiplex microsphere-based assay. FRNT=focus reduction neutralisation test. MFI=mean fluorescence intensity. ZIKV=Zika virus.

Considering the predominance of inapparent primary infections, serotyping these infections is critical to evaluate the epidemiological burden and virulence associated with each serotype. Thus, we evaluated the performance of the EDIII-MMBA to serotype primary infections. For this analysis, we used the evaluation set, which consisted of 156 late convalescent samples from the annual sample collection (86 inapparent infections and 70 symptomatic infections). Exceptional performance (100% sensitivity, specificity, and accuracy) and concordance were observed when evaluated against both RT-PCR and FRNT for DENV1–4 and ZIKV (table, figure 2).

**Table:** EDIII-MMBA performance

		**DENV1**	**DENV2**	**DENV3**	**DENV4**	**ZIKV**
**RT-PCR *vs* EDIII-MMBA**						
Total number of samples evaluated		70	70	70	70	70
Gold standard results						
	RT-PCR positive	16	13	20	6	15
	RT-PCR negative	54	57	50	64	55
EDIII-MMBA results						
	True positive	16	13	20	6	15
	False positive	0	0	0	0	0
	False negative	0	0	0	0	0
	True negative	54	57	50	64	55
Sensitivity (95% CI)		100% (79·4–100)	100% (75·3–100)	100% (83·2–100)	100% (54·1–100)	100% (78·2–100)
Specificity (95% CI)		100% (93·4–100)	100% (93·7–100)	100% (92·9–100)	100% (94·4–100)	100% (93·5–100)
Diagnostic accuracy (95% CI)		100% (94·9–100)	100% (94·9–100)	100% (94·9–100)	100% (94·9–100)	100% (94·9–100)
**FRNT *vs* EDIII-MMBA**						
Total number of samples evaluated		86	86	86	0	86
Gold standard results						
	FRNT positive	38	19	19	..	10
	FRNT negative	48	67	67	..	76
EDIII-MMBA results						
	True positive	38	19	19	..	10
	False positive	0	0	0	..	0
	False negative	0	0	0	..	0
	True negative	48	67	67	..	76
Sensitivity (95% CI)		100% (90·7–100)	100% (82·4–100)	100% (82·4–100)	..	100% (69·2–100)
Specificity (95% CI)		100% (92·6–100)	100% (94·6–100)	100% (94·6–100)	..	100% (95·3–100)
Diagnostic accuracy (95% CI)		100% (95·8–100)	100% (95·8–100)	100% (95·8–100)	..	100% (95·8–100)

DENV=dengue virus. EDIII-MMBA=envelope domain III multiplex microsphere-based assay. FRNT=focus reduction neutralisation test. ZIKV=Zika virus.

The EDIII-MMBA was conducted on 574 (46%) of the 1244 primary inapparent infections recorded in the PDCS between 2004 and 2022. These 574 cases comprised samples from 478 (87%) individuals from the random selection of individuals with primary DENV infection, plus 76 (13%) inapparent samples (excluding ten ZIKV infections) from the evaluation subset. Of the 574 samples, 425 (74%) were successfully serotyped. 35 (6%) of 574 infections were excluded due to either invalid serotyping (n=21) or ZIKV infection (n=14; appendix 2 p 22), resulting in 1591 primary DENV infections out of the 1626 total primary infections recorded by iELISA and RT-PCR in the study period. Anti-EDIII antibody levels below the detection limit (1:50) were observed in 114 (20%) samples. This discrepancy might be due to the difference in the type of antibody measured (anti-EDIII IgG), compared with the iELISA used initially to detect infection (total Ig primarily directed towards anti-pr and anti-E fusion loop antibodies).^3^ Using the total number of samples screened, we performed an analysis of the capacity of the EDIII-MMBA to provide valid serotyping of primary infection samples as detected by iELISA (n=564). Including the 114 samples with no detectable anti-EDIII antibodies and the 21 samples with invalid serotyping, the sensitivity of the EDIII-MMBA was 76·5% (95% CI 72·8–79·9), compared with 95·4% (93·1–97·2) when only participants with anti-EDIII antibodies were included (n=460). The breakdown of serotyped and non-serotyped infections, along with a flowchart illustrating the screening and serotyping process, are provided in appendix 2 (pp 9, 22). Missing serotype data on the remaining inapparent and symptomatic infections, including those with no detectable EDIII, were imputed using results of EDIII-MMBA and RT-PCR, respectively (appendix 2 p 23). The convergence of the final imputation models was assessed using trace plots, which indicated robust performance with no discernible trends in the final iterations (appendix 2 pp 24–26). Our final imputation models were validated and showed high agreement with the experimentally serotyped data, with an overall accuracy of 0·89 (95% CI 0·85–0·92) for symptomatic infection and 0·78 (0·74–0·82) for inapparent infection (appendix 2 pp 10–12).

**Figure 3:**
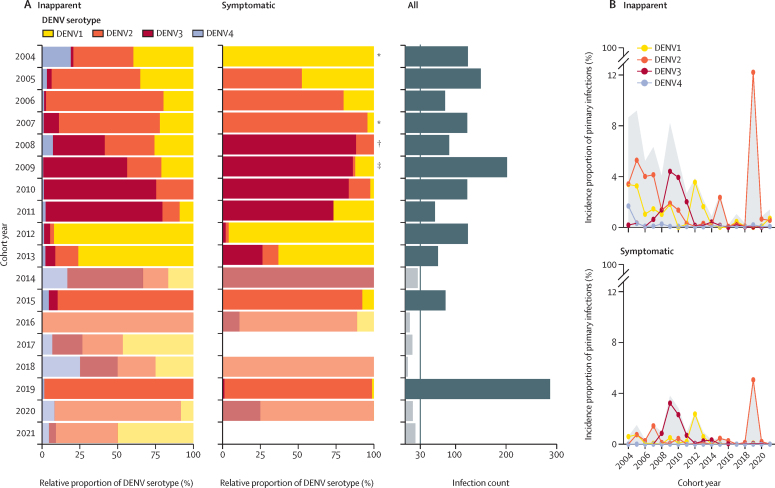
Serotype distribution in primary symptomatic and inapparent infections by serotype from 2004 to 2022 in the PDCS (A) Pooled relative distribution of DENV serotypes circulating yearly in inapparent and symptomatic primary DENV infections across multiple imputations. Transparency was added in years with infection count <30. The grey panel shows the total incidence of primary DENV infections. (B) Temporal dynamics of the percent of primary DENV infections by infection outcome (shown in upper and lower panels) and serotype across imputations. The grey area covers the pooled incidence proportions of overall primary DENV infections by infection outcome. The x-axes show the cohort year; cohort year 2021 ended in March, 2022. DENV=dengue virus. PDCS=Pediatric Dengue Cohort Study. *p<0·05. †p<0·01. ‡p<0·001.

Throughout the study period, DENV serotypes 1, 2, and 3 dominated, representing 96% of total primary DENV infections (1527/1591), reflecting the serotypes circulating in the country.^13^ In contrast, DENV4 circulation was very low and was exclusively detected in inapparent infections. The analysis of primary infections revealed distinct serotype distributions, with substantial differences observed between the proportions of symptomatic versus inapparent infections caused by each serotype every year (figure 3A). For instance, in 2004, DENV1 was the only serotype detected in symptomatic cases, whereas all four serotypes circulated within the inapparent infection fraction. During 2008–10, DENV3 was dominant in symptomatic cases but exhibited lower relative prevalence within the inapparent fraction. Significant within-year variations in serotype distribution were observed between the symptomatic and inapparent infection fractions, particularly in 2004 (p=0·011), 2007 (p=0·021), 2008 (p=0·0025), and 2009 (p=0·0005; figure 3A, appendix 2 p 13). These variations and temporal dynamics were consistent across experimentally serotyped and imputed datasets (figure 3A and appendix 2 p 23).

Subsequent stratification of the cumulative incidence proportions of inapparent and symptomatic infections by serotype (figure 3B) further accentuates the pattern observed initially: whereas symptomatic infections tend to be dominated by a single serotype, the DENV landscape unveiled by inapparent infections is more complex, with co-circulation of multiple DENV serotypes, including serotypes undetected in symptomatic surveillance, in multiple years.

**Figure 4:**
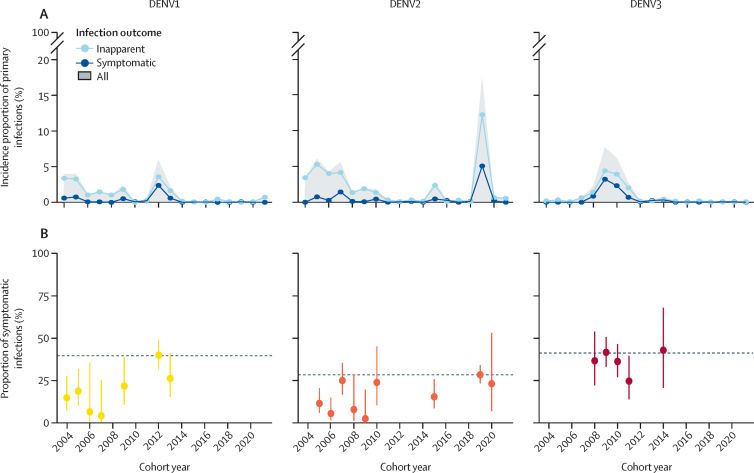
Temporal dynamics of symptomatic, inapparent, and overall primary DENV infections by serotype from 2004 to 2022 in the PDCS (A) Pooled percentage of primary DENV infections by infection outcome and serotype, as indicated in left, middle, and right panels across imputations. The grey area covers the pooled incidence proportions of overall primary DENV infections by serotype. (B) Proportion of symptomatic infections among total primary DENV infections by serotype (indicated in left, middle, and right panels). This analysis excludes years when no symptomatic infections or <10 primary infections were reported. This is the result of the pooled predicted marginal probabilities of disease given infection from logistic regressions adjusting for year, compared with reference years (dashed line) of the most intense outbreak (DENV1: 2012, DENV2: 2019, DENV3: 2009). The x-axes show the cohort year; cohort year 2021 ended in March, 2022. DENV=dengue virus. PDCS=Pediatric Dengue Cohort Study.

DENV2 demonstrated the most extended circulation period and the highest overall incidence in our study compared with DENV1 and DENV3 (appendix 2 p 14). Nevertheless, analysing the cumulative annual incidence proportion of primary symptomatic and inapparent infections per serotype highlighted dynamic shifts in the dominance of DENV serotypes. While DENV2 dominated the initial years of the study, this dominance transitioned to DENV3 in 2009–10 and subsequently shifted to DENV1 during the 2012–13 period, before DENV2 resurgence in 2019 (figure 4A, appendix 2 p 15). A marked change in DENV2 incidence proportion was particularly notable in 2019, with 17% (95% CI 15–19) of the DENV-naive population experiencing primary DENV2 infections compared with only 4% (3–5) in 2006 (figure 4A, appendix 2 pp 15, 27). The incidence proportion of primary DENV3 infections was particularly high between 2008 and 2011, reaching up to 7% (6–9) of the DENV-naive population at its peak, whereas for DENV1, incidence peaked in 2012 (6%, 5–7). Adjusting for clustering by individual did not change these results, as the total variability is captured by epidemic year (appendix 2 pp 16–17, 27).

Analysing the proportion of symptomatic infections among total infections per serotype over time revealed considerable yearly fluctuations. Excluding years when no symptomatic infections were found and years with fewer than ten primary infections per serotype, the proportion of DENV1 symptomatic infections among DENV1 total infections ranged from 7% (95% CI 1–35) in 2006 to a peak of 40% (32–49) in 2012; DENV2 ranged from 6% (2–16) in 2006 to 29% (24–35) in 2019, and DENV3 ranged from 25% (15–40) in 2011 to 44% (22–69) in 2014 (figure 4B, appendix 2 p 15). Notably, at the serotype level, years of the most intense outbreaks coincided with the highest proportion of symptomatic infections for all serotypes. To evaluate differences in virulence over time by serotype, we also report the values in pooled odds ratio form (appendix 2 p 18).

**Figure 5:**
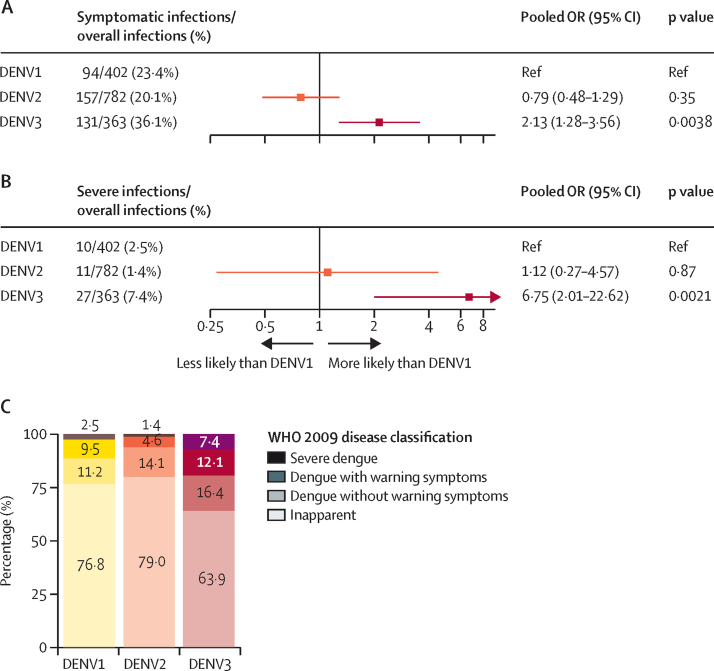
Spectrum of disease of primary DENV infections (A) Pooled counts of symptomatic and overall primary DENV infections with crude percentages of symptomatic primary DENV infections by DENV serotype and pooled ORs adjusting by year showing the association between dengue case and serotype among primary DENV infections. (B) Pooled counts of severe and overall primary DENV infections with crude percentages of severe primary DENV infections by DENV serotype and pooled ORs adjusting by year showing the association between dengue case and serotype among primary DENV infections. (C) Spectrum of infection outcome and disease severity according to the 2009 classification as the crude pooled percentages of primary DENV infections. DENV=dengue virus. OR=odds ratio.

Further investigation into the disease spectrum of primary infections underscored stark differences by serotype. Analysing symptomatic versus inapparent and severe versus non-severe outcomes, DENV3 demonstrated significantly higher likelihood of symptomatic (pooled odds ratio 2·13, 95% CI 1·28–3·56) and severe (6·75, 2·01–22·62) infections, compared with DENV1 adjusting by epidemic year, whereas DENV2 did not show significant differences in either analysis compared with DENV1 (figure 5A, B). Such results were consistent across all the sensitivity analyses performed (appendix 2 pp 19–20).

Lastly, we summarised the spectrum of disease observed in primary infections caused by the three major DENV serotypes that circulated in Nicaragua between 2004 and 2022 by analysing the frequency of inapparent, symptomatic, and severe DENV infections classified according to the WHO guidelines from 2009 (figure 5C). Although we found that the majority of primary infections remained inapparent, symptomatic infection was observed in 23·2% (95% CI 19·2–27·8), 20·1% (17·4–23·2), and 36·1% (31·1–41·4) of DENV1, DENV2, and DENV3 infections respectively. Among total primary infections, severe disease was observed in 2·5% (1·3–4·6) of DENV1, 1·4% (0·8–2·6) of DENV2, and 7·4% (5·1–10·6) of DENV3 infections, further highlighting the variability in infection outcomes associated with each serotype (appendix 2 p 21).

## Discussion

Our study provides novel insights into serotype-specific epidemiological patterns and disease outcomes of primary DENV infections in Nicaragua by revealing the hidden contribution of inapparent infections. The EDIII multiplex microsphere-based assay has been previously evaluated to detect any prior ZIKV or DENV infection, showing high sensitivity and specificity.^11^ Here we expanded and evaluated its use for serotyping primary DENV infections, which demonstrated excellent performance when evaluated against the gold standard FRNT and RT-PCR assays. We believe that this high accuracy can be attributed to the study design and to parameters inherent to the PDCS and the EDIII antigen (detailed in appendix 2 p 29). This major methodological advancement offers a scalable and efficient alternative to the labour-intensive neutralisation assays traditionally used to serotype infection. Compared with neutralisation assays such as the FRNT, the microsphere-based assays are easier to standardise across laboratories and can be performed without the need for handling infectious materials under Biosafety Level 2 conditions.

The majority of primary infections within our cohort were inapparent (76%), aligning with existing literature that suggests a high prevalence of inapparent and subclinical infection across dengue-endemic regions.^13^, ^14^, ^15^, ^16^ However, the proportion of inapparent infections greatly fluctuates across years. The characterisation of these infections allowed us to examine the serotype-specific proportion of primary inapparent infections and their temporal variability for the first time. Importantly, our study shows that while a single serotype often dominates symptomatic primary infections in any given period, the landscape of inapparent infections in Nicaragua is far more complex, revealing the co-circulation of multiple serotypes and serotype circulation undetected in symptomatic cases across multiple years. This observation suggests that the epidemiological footprint of DENV is broader and more nuanced than what is captured by monitoring symptomatic cases alone, whenever there is co-circulation of serotypes or hyperendemicity. This finding underscores the utility of continuous and comprehensive surveillance systems that capture both symptomatic and inapparent infections to accurately assess the epidemiological burden of DENV and inform public health interventions.

Temporal variations in the incidence and proportion of symptomatic primary infections further highlighted the evolving landscape of dengue in Nicaragua. Notably, we observed an increase in virulence of DENV1 over time, with more symptomatic primary infection in 2012 compared with 2004–06. Furthermore, DENV2 infectivity surged in 2019, with 17% of the DENV-naive population experiencing primary infections versus 5% in 2006. These changes could potentially be due to strain differences over time. Although our study does not directly link these observations to specific viral genetic variation, this hypothesis is supported by previous studies showing lineage shifts in DENV1 and DENV2 within our cohort (appendix 2 p 28).^17^, ^18^, ^19^ Furthermore, the level of population-level susceptibility to different serotypes, as well as changes in host or environmental factors might also influence infection dynamics and outcomes. For instance, the extensive circulation of ZIKV in 2016 likely enhanced the DENV2 epidemic in 2019.^5^, ^6^, ^20^, ^21^, ^22^ Additionally, increases in comorbidities (eg, the rise in prevalence of obesity) in our cohort might also influence dengue disease burden.^23^ Finally, dengue cohort studies have reported large fluctuations in the yearly proportion of symptomatic infections and have determined that the predominant circulating serotype is one of the main factors driving this fluctuation.^8^, ^24^

By dissecting the full disease spectrum associated with primary DENV infection, our study revealed stark serotype-specific differences in infection outcome. Specifically, our finding supports and extends existing literature by demonstrating that DENV3 primary infections were not only significantly more severe,^25^, ^26^, ^27^ but also significantly more symptomatic compared with DENV1 and DENV2. For instance, a study of schoolchildren in Thailand found that schools with high DENV3 circulation were associated with lower detection of inapparent infections.^24^ Another study in Nicaragua found that among symptomatic infections, more severe primary infections are caused by DENV3 than by DENV1 or DENV2.^27^ Here, we expand on these results by taking the full spectrum of primary infections into consideration, including inapparent infections. Furthermore, our study shows that the low rate of symptomatic primary DENV2 infections in Nicaragua is not due to a scarcity of primary infections but rather due to a high rate of inapparent infections. Also, it is important to note that while severe dengue is more prevalent during secondary infections, it also occurs in primary infections, particularly with DENV3 and DENV1.^27^, ^28^, ^29^, ^30^ Substantial morbidity and mortality can be attributed to primary infections; for example, more than 50% of severe cases and fatalities in a study in India^31^ and 23% of patients hospitalised with dengue in a study in Mexico^32^ were attributed to primary infections. Here, we also found a sizeable amount of severe infections among primary cases, being the highest for DENV3. Of note, the genotype circulating in Nicaragua from 2008 to 2014 was DENV3 GIII C.2. Altogether, these studies show that primary infections contribute substantially to disease burden. Thus, safe and effective vaccines for DENV-naive individuals are warranted.

This study has several strengths. First, we expanded the use of a new simpler, high-throughput method for serotyping inapparent primary infections. Second, we analysed 17 years of data based on a robust cohort study design that enables investigation of temporal dynamics of multiple DENV serotypes in a consistent, single setting with a large sample size. The study design captures inapparent, mild, and severe DENV infections, offering a comprehensive view of the disease burden that refines our understanding of DENV epidemiology. Furthermore, while serotyping inapparent primary infections is not unique to our study, most studies are cross-sectional,^33^, ^34^, ^35^ reporting crude seroprevalence of a given serotype. Here, we report the evolution of the incidence of primary infection by serotype over time.

However, our study has several limitations, including minimal DENV4 circulation during the study timeframe. Nonetheless, among primary infections, we predict that DENV4 has a similar spectrum of symptomatic versus inapparent infection outcome compared with DENV2, as evidenced in the literature.^25^ The emergence of ZIKV might have impacted the DENV epidemiology in Nicaragua, which would affect our findings after 2016, especially for DENV2. Yet, when restricting our analyses from 2004 to 2015, our findings were similar. As our findings reflect the serotype distribution in Nicaragua, the generalisability of our findings might be somewhat limited by the regional focus and the genotype of local DENV strains (appendix 2 p 21), which could have distinct virulence and infectivity. Nevertheless, we believe that our findings provide a robust rationale for future research in different settings to assess the epidemiology of other DENV genotypes and broader implications of our findings. The random selection of inapparent samples for EDIII serotyping likely mitigated selection bias, providing an unbiased estimate of serotype distribution among inapparent infections, but our imputation model assumes such data to be missing at random, dependent on time and space; deviations from this assumption could bias our findings. Our final imputation models were valid, except for DENV4, which accounted for only 2% of the inapparent infections. Our results remained robust when excluding DENV4 infections from our imputation models and across other sensitivity analyses. Finally, although we adjusted for age and sex in the imputation and main endpoint models, we cannot rule out residual confounding.

In summary, using a high-throughput serotyping method to analyse inapparent primary DENV infections in a cohort over 17 years, our study enriches the epidemiological data landscape with unprecedented detail. Our findings reveal significant differences in the epidemiological profile of symptomatic and inapparent DENV infections and show serotype-specific patterns and disease spectra. The stark differences in disease outcome by serotype, particularly the symptomatic and severe nature of DENV3 infections versus others, highlight the need for balanced and effective vaccines across serotypes that provide protection even for those without previous DENV exposure. These insights are also important for refining existing models of DENV transmission and mark an advancement in our understanding of the serotype-specific risks of infection outcome, which allows us to better predict outbreaks and hospital admission, understand DENV transmission dynamics, and inform vaccine development.

### Contributors

### Data sharing

All relevant data supporting the findings of this study are included within the manuscript or appendix 2. Normalised EDIII-MMBA values from our evaluation set are accessible in appendix 3. We kindly request that researchers who use the data provided for their own studies acknowledge this by citing this paper. Additional datasets generated or analysed during the current study are available upon reasonable request, following the protocol approved by the Institutional Review Board for the PDCS. Researchers who wish to access the additional data are encouraged to submit a formal request to EH or the Committee for the Protection of Human Subjects at the University of California, Berkeley. In accordance with ethical guidelines and to ensure the proper use of the data, all requests will be reviewed and approved on a case-by-case basis. The associated code for this study is available for reference on Zenodo (https://doi.org/10.5281/zenodo.12349676).

## Declaration of interests

AG has received institutional payments from Flu Lab and Open Philanthropy, personal honoraria from Hope College and La Jolla Institute of Immunology, payments for expert testimony from Berman and Simmons, and travel support from the Gates Foundation and National Institutes of Health (NIH), and has an advisory role with Janssen Pharmaceuticals. All other authors declare no competing interests.

## Supplementary Materials

Spanish translation of the abstract
