# Supplementary material for: Serotype-specific epidemiological patterns of inapparent versus symptomatic primary dengue virus infections: a 17-year cohort study in Nicaragua
Source: Lancet Infect Dis. 2025 Mar;25(3):346–56. doi: 10.1016/S1473-3099(24)00566-8 (PMC11864988; doi:10.1016/S1473-3099(24)00566-8)
Supplement: Spanish translation of the abstract [file mmc1.pdf]

THE LANCET
Infectious Diseases

Supplementary appendix 1

This translation in Spanish was submitted by the authors and we reproduce it as supplied. It has not been peer reviewed. *The Lancet*'s editorial processes have only been applied to the original in English, which should serve as reference for this manuscript.

Los autores nos proporcionaron esta traducción al español y la reproducimos tal como nos fue entregada. No la hemos revisado. Los procesos editoriales de *The Lancet* se han aplicado únicamente al original en inglés, que debe servir de referencia para este manuscrito.

Supplement to: Bos S, Zambrana JV, Duarte E, et al. Serotype-specific epidemiological patterns of inapparent versus symptomatic primary dengue virus infections: a 17-year cohort study in Nicaragua. *Lancet Infect Dis* 2024; published online Oct 25. https://doi.org/10.1016/S1473-3099(24)00566-8.

Patrones epidemiológicos específicos por serotipo de infecciones primarias inapparentes versus sintomáticas del virus del dengue: un estudio de cohorte de 17 años en Nicaragua

Sandra Bos*, José Victor Zambrana*, Elias Duarte, Aaron L Graber, Julia Huffaker, Carlos Montenegro, Lakshmanane Premkumar, Aubree Gordon, Guillermina Kuan, Angel Balmaseda, Eva Harris
*Contribuyeron igualmente

División de Enfermedades Infecciosas y Vacunología, Escuela de Salud Pública, Universidad de California, Berkeley, Berkeley, California, EEUU (S Bos PhD, E Duarte BS, A L Graber BA, J Huffaker BS, Prof E Harris PhD); Instituto de Ciencias Sostenibles, Managua, Nicaragua (J V Zambrana MPH, C Montenegro BS, G Kuan MD, A Balmaseda MD); Departamento de Epidemiología, Escuela de Salud Pública, Universidad de Michigan, Ann Arbor, Michigan, EEUU (J V Zambrana, Prof A Gordon PhD); Departamento de Microbiología e Inmunología, Escuela de Medicina de la Universidad de Carolina del Norte, Chapel Hill, North Carolina, EEUU (L Premkumar PhD); Centro de Salud Sócrates Flores Vivas, Ministerio de Salud, Managua, Nicaragua (G Kuan); Laboratorio Nacional de Virología, Centro Nacional de Diagnóstico y Referencia, Ministerio de Salud, Managua, Nicaragua (A Balmaseda)

Correspondencia a:
Prof. Eva Harris, División de Enfermedades Infecciosas y Vacunología, Escuela de Salud Pública, Universidad de California, Berkeley, Berkeley, CA 94720-3370, EEUU
eharris@berkeley.edu

Resumen
Antecedentes: El dengue es la enfermedad viral transmitida por mosquitos más prevalente y un importante problema de salud pública a nivel mundial. La mayoría de las infecciones primarias con los cuatro serotipos del virus del dengue (DENV1–4) son inaparentes; sin embargo, se desconoce si la distribución de infecciones sintomáticas versus inaparentes varía según el serotipo. Aquí presentamos (1) la evaluación de un ensayo multiplex del dominio III de la envoltura de DENV1-4 basado en microesferas (EDIII-MMBA) para serotipificar infecciones primarias inaparentes y (2) su aplicación aprovechando 17 años de recolección de muestras prospectivas del Estudio de Cohorte Pediátrica del Dengue en Nicaragua (PDCS).

Métodos: Analizamos infecciones primarias por DENV en el PDCS desde 2004 hasta 2022 detectadas por ELISA de inhibición (iELISA) o RT-PCR. Primero, evaluamos el desempeño del EDIII-MMBA con muestras caracterizadas por RT-PCR o prueba de neutralización por reducción de focos. Luego, analizamos un subconjunto del total de infecciones primarias inaparentes por DENV en el PDCS con el EDIII-MMBA para evaluar la epidemiología de las infecciones inaparentes. Las infecciones restantes se infirieron mediante imputación estocástica, teniendo en cuenta el año y el vecindario. La incidencia de infección y el porcentaje de infecciones inaparentes, sintomáticas y graves se analizaron por serotipo.

Resultados: Entre el 30 de agosto de 2004 y el 10 de marzo de 2022, un total de 5931 participantes naïve a DENV fueron seguidos en el PDCS. Hubo 1626 infecciones primarias (382 sintomáticas, 1244 inaparentes) detectadas por iELISA o RT-PCR durante el período de estudio. El EDIII-MMBA demostró una excelente precisión general (100%, IC 95% 95.8-100) para serotipificar infecciones primarias inaparentes por DENV cuando se le evaluó frente a métodos de serotipificación de referencia. De las 1244

infecciones inaparentes, analizamos 574 (46%) utilizando el EDIII-MMBA. Encontramos que la mayoría de las infecciones primarias fueron inaparentes, con DENV3 exhibiendo la mayor probabilidad de infecciones primarias sintomáticas (razón de probabilidades agrupada en comparación con DENV1: 2.13, IC 95% 1.28-3.56) y graves (6.75, 2.01-22.62), mientras que DENV2 fue similar a DENV1 en ambos análisis. Se observó una variación considerable intra-anual y entre años en la distribución de serotipos entre infecciones sintomáticas e inaparentes y se demostró la circulación de serotipos no detectados en casos sintomáticos en varios años.

Interpretación: Nuestro estudio indica que la vigilancia de casos sesga la percepción del impacto epidemiológico del DENV. Revelamos un patrón más complejo e intrincado de distribución de serotipos en infecciones inaparentes. Las diferencias sustanciales en los resultados de infección según el serotipo enfatizan la necesidad de vacunas con inmunogenicidad y eficacia equilibradas entre los serotipos.

Financiación: Instituto Nacional de Alergias y Enfermedades Infecciosas (Institutos Nacionales de Salud) y Fundación Bill y Melinda Gates.
