# Supplementary material for: Serotype-specific epidemiological patterns of inapparent versus symptomatic primary dengue virus infections: a 17-year cohort study in Nicaragua
Source: Lancet Infect Dis. 2025 Mar;25(3):346–56. doi: 10.1016/S1473-3099(24)00566-8 (PMC11864988; doi:10.1016/S1473-3099(24)00566-8)
Supplement: Supplementary appendix 2 [file mmc2.pdf]

THE LANCET
Infectious Diseases

Supplementary appendix 2

This appendix formed part of the original submission and has been peer reviewed. We post it as supplied by the authors.

Supplement to: Bos S, Zambrana JV, Duarte E, et al. Serotype-specific epidemiological patterns of inapparent versus symptomatic primary dengue virus infections: a 17-year cohort study in Nicaragua. *Lancet Infect Dis* 2024; published online Oct 25. https://doi.org/10.1016/S1473-3099(24)00566-8.

Supplementary Appendix

Table of contents

Supplementary Methods **2**
Ethics statement 2
The Pediatric Dengue Cohort Study and Participants 2
Case and inapparent infection definition 2
Severity classification as per the 2009 WHO classification 3
EDIII-MMBA 3
Neutralization assay 4
DENV iELISA 4
Typing of primary inapparent infection based on EDIII-MMBA 5
Evaluation of the EDIII-MMBA 5
Covariate definition and relationship between variables 6
Sensitivity analyses 6
Evaluation of the final imputation models 7
Supplementary figures and tables **8**
Table S1. Count of naïve participants and primary DENV infections per year 8
Table S2. Breakdown of serotyped and non-serotyped inapparent and symptomatic primary DENV infections 9
Table S3. Imputation model performance on inapparent infections based on the experimentally serotyped data 10
Table S4. Imputation model performance on symptomatic infections based on the experimentally serotyped data 10
Table S5. Actual vs predicted inapparent DENV infections based only on the experimentally serotyped data 11
Table S6. Actual vs predicted symptomatic DENV infections based only on the experimentally serotyped data 12
Table S7. Significance of fisher tests 13
Table S8. Overall incidence of primary DENV infections by serotype. 14
Table S9. Risk of dengue infections and percent symptomatic given primary infection by dengue serotype over time, based on model predictions across imputations excluding years with low symptomatic circulation (n < 10). 15
Table S10. Overall incidence of primary DENV infections by serotype accounting for clustering. 16
Table S11. Risk of dengue infections and percent symptomatic given primary infection by dengue serotype over time, based on model predictions across imputations excluding years with low symptomatic circulation (n < 10) accounting for clustering. 17
Table S12. Odds ratios of symptomatic primary infection given DENV1 infection over time 18
Table S13. Odds ratios of symptomatic primary infection given DENV2 infection over time 18
Table S14. Odds ratios of symptomatic primary infection given DENV3 infection over time 18
Table S15. Sensitivity analyses 19
Table S16. Outcome of primary infections 21
Table S17. Genotype of Nicaraguan DENV serotypes. 21
Figure S1. Flowchart of the screening and serotyping process to study the epidemiology of primary DENV infections in the PDCS 22
Figure S2. Distribution of serotyped, non-serotyped, and imputed symptomatic and inapparent primary DENV from 2004 to 2021 in the PDCS 23

Figure S3. Trace plots for the final imputation models 24
Figure S4. Trace plots for the final imputation framework based on only 10 simulations 25
Figure S5. Trace plots for the final imputation framework based on only 10 simulations and 30 iterations 26
Figure S6. Temporal dynamics of overall primary DENV infections by serotype from 2004 to 2021 in the PDCS 27
Figure S7. Temporal shift in Nicaraguan DENV2 lineages 28
Supplementary Discussion 29
Supplementary References 30

Supplementary Methods

Ethics statement

The human subjects protocol for the Pediatric Dengue Cohort Study (PDCS) was reviewed and approved by the Institutional Review Boards (IRB) of the University of California, Berkeley (2010-09-2245), the University of Michigan (HUM00091606), and the Nicaraguan Ministry of Health (CIRE-09/03/07-008). Parents or legal guardians of all subjects provided written informed consent, and subjects 6 years of age and older provided assent.

The Pediatric Dengue Cohort Study and Participants

The PDCS is an ongoing prospective study initiated in 2004 and carried out at the Sócrates Flores Vivas Health Center (HCSFV), located in District 2 of Managua, Nicaragua.[1] Located adjacent to Lake Xoxitlan, District 2 encompasses neighborhoods spanning a spectrum of low to middle socioeconomic status. The HCSFV serves ~62,000 residents across 18 neighborhoods within District 2.[2] The illiteracy rate in this area is approximately 7%, and over 90% of the households have access to running water and a sewage system. All residents have access to electricity.[3]

The PDCS tracks approximately 4,000 active participants yearly and has followed ~9,800 children since its inception. To preserve the original age distribution of the cohort, approximately 300 two-year-old children, along with children aged 3-11 years as needed, are enrolled annually. Noteworthy adjustments to the age limit were implemented in 2018, 2019, and 2022, extending the eligibility to 15, 16, and 17 years, respectively. Participants benefit from free, round-the-clock medical care provided by study physicians at the HCSFV. In cases requiring hospitalization, study staff facilitate patient transfer to the study hospital, the National Pediatric Reference Hospital (Hospital Infantil Manuel de Jesús Rivera). Children presenting with fever and since July 2016, rash, are screened for signs and symptoms of dengue, Zika, and chikungunya. Symptomatic infections are captured through passive surveillance, complemented with periodic home visits. For each suspected case, both acute (0-5 days post-symptom onset) and convalescent samples (14-21 days) are collected to confirm infection. Approximately 97% of cases present to the HCSFV within the first three days of illness, and convalescent samples are collected from 94% of cases.[1] Additionally, the study collects annual healthy blood samples from all participants in March-April (previously in July before 2011), which enable the detection of inapparent arboviral infections.[4]

A series of annual surveys are administered verbally, and answers are recorded on smartphones or tablet computers using custom-built software. Two questionnaires are administered, one for every household and one for every participant. The participant questionnaire covers demographic information such as biological sex (with no genetic confirmation), age, and education level. The household questionnaire includes questions on assets and conditions of the home.

Case and inapparent infection definition

Upon medical examination, children enrolled in the study are categorized into A, B, C, or D cases, as follows: A: individuals meeting the 1997 or 2009 World Health Organization (WHO) case definition for dengue; B: those with undifferentiated fever of undefined origin; C: children with an acute febrile illness identified as a disease other than dengue, chikungunya, or Zika; D: individuals with other conditions (including rash) that do not include fever. A comprehensive array of clinical and laboratory information, encompassing 189 different variables4 is collected for each participant. This includes vital signs, temperature, musculoskeletal pain, respiratory symptoms, gastrointestinal symptoms, indicators of dehydration, rashes, and other skin anomalies. Cases categorized as A and B undergo testing for acute

DENV and, since 2014, chikungunya virus (CHIKV) infection. Since 2015, cases meeting the criteria of A, B, or D (rash without fever) are also tested for acute Zika virus (ZIKV) infection.

Detection of cases

Acute samples are examined using the ZCD multiplex real-time reverse transcriptase-PCR (rRT-PCR) assay for DENV, ZIKV, and CHIKV;5 before the introduction of ZIKV in Nicaragua, different RT-PCR methods were used.6,7 DENV-positive samples are then serotyped by multiplex rRT-PCR, and a subset undergo virus isolation in C6/36 cells.7,8 Evaluation of paired acute-phase and convalescent-phase samples is conducted to detect seroconversion of IgM antibodies, indicating current infection. This is done using separate in-house DENV and ZIKV IgM capture (MAC)-ELISA assays.9,10 DENV inhibition ELISA (iELISA) and ZIKV iELISA assays are used to measure total anti-DENV and -ZIKV antibodies, respectively, and to detect seroconversion or a ≥4-fold increase in antibody titer. Additionally, a computer algorithm assists in identifying Zika cases from dengue cases based on the results of the aforementioned serological tests.11 Those with serotyping by RT-PCR that was inconclusive due to unclear results or low viremia, which were positive by either MAC ELISA or iELISA were considered DENV serology cases.

Inapparent infections

Before 2016, inapparent DENV infections were identified if a seroconversion or a ≥4-fold increase in antibody titer between annual samples was observed, as measured by DENV iELISA, in the absence of any observed febrile episode identified as a dengue case in the intervening year.12 Following the 2016 Zika epidemic, inapparent DENV infections were defined as seroconversion or a ≥4-fold rise in DENV iELISA titer in absence of ZIKV seroconversion (measured by ZIKV NS1 BOB ELISA or ZIKV iELISA) or an observed dengue, Zika, or flavivirus case in that year.9 Seroconversion by ZIKV NS1 BOB ELISA and/or seroconversion or >4-fold rise by ZIKV iELISA was used to detect inapparent ZIKV infections.

Severity classification as per the 2009 WHO classification

Dengue without Warning Signs (DwoWS): A laboratory-confirmed dengue case manifesting fever and two of the following clinical features: nausea/vomiting, rash, aches and pains, positive tourniquet test, leukopenia.

Dengue with Warning Signs (DwWS): A laboratory-confirmed dengue case manifesting symptoms such as abdominal pain, persistent vomiting, fluid accumulation, lethargy, mucosal bleeding, liver enlargement, or a rise in hematocrit concurrent with a decrease in platelet count.[13]

Severe Dengue (SD): A laboratory-confirmed dengue case featuring severe bleeding, significant plasma leakage leading to shock, fluid accumulation resulting in respiratory distress, and/or organ failure. Organ involvement was indicated by liver ALT or AST levels of ≥1,000, impaired consciousness, or heart or other organ failures.

EDIII-MMBA

Recombinant envelope domain III (EDIII) proteins of DENV1-4 and ZIKV (gift from Lakshmanane Premkumar, University of North Carolina, Chapel Hill) were site-specifically biotinylated, conjugated to avidin-coated MagPlex Luminex beads and combined. Immune complexes were formed in 384-well plates by mixing appropriately diluted plasma (four 4-fold dilutions starting at 1:50) with antigen-coupled

microspheres (500 microsphere/antigen/well) for 90 minutes at 37°C, shaking at 1200 rpm. After incubation, plates were washed using an automatic magnetic washer (Tecan Hydrospeed) with phosphate-buffered saline (PBS) containing 0.1% bovine serum albumin (BSA) and 0.02% Tween 20. Antigen-specific IgG binding was detected using phycoerythrin (PE)-coupled mouse anti-human total IgG (SouthernBiotech). Median fluorescence intensity (MFI) was measured using an iQue3 (Intellicyt) instrument set to acquire at least 50 beads per bead region. DENV-naïve human serum (NHS) was run in triplicate on each plate. Positive signal was defined as an MFI greater than the average MFI of NHS + 3 SD at the highest dilution (1:50). Negative signal (e.g., absence of EDIII antibody) was defined as an MFI lower than the average MFI of NHS + 3 SD at the highest dilution (1:50).

Neutralization assay

The focus reduction neutralization test (FRNT) assay was conducted using Vero cells and clinical isolates from Nicaragua (DENV1 5575.10a1SPD1, DENV2 8891.12a1SPD2, DENV3 6629.10a1SPD3, and ZIKV Nica2-16) or DENV4 Sri Lanka 92 strain (NCBI KJ160504.1) as previously described.[14] Briefly, cells were seeded in a 96-well plate one day before infection. Serum/plasma were serially diluted and mixed with an equal inoculum of DENV at a volume ratio of 1:1 to form immune complexes. The virus-antibody mixture was incubated for 1 hour at 37°C in 5% CO_2. Cell substrate growth medium was removed, and the virus-antibody mixture was added to the cells and incubated for 1 hour at 37°C in 5% CO_2. Subsequently, a 0.6% carboxymethylcellulose overlay was added, and the plates were incubated for 48 hours. Foci were developed and counted using an CTL Immunospot analyzer (Cellular Technology, Ltd.). Foci counts were fitted using a variable slope dose response curve using Graphpad, and neutralizing antibody titer (NT_{50}) values were calculated with constrained top and bottom values of 100 and 0, respectively. Percent relative infection was calculated as a ratio of foci counts in each serial dilution to the foci counts from the final serial dilution of each sample. All samples and mAbs were run in duplicate with reported values required to have an $R^2 > 0.85$ and a Hill slope > 0.5. The serotype responsible for the inapparent infection was identified as the serotype to which the serum showed the highest neutralization.

DENV iELISA

The inhibition ELISA (iELISA) protocol,[12] adapted from the method by Fernández and Vázquez[15], is a diagnostic assay to detect antibodies against DENV in serum samples. The protocol involves coating 96-well polystyrene plates with anti-DENV polyclonal human IgG and incubating overnight at room temperature. After washing, the wells are blocked with BSA, then incubated at 37°C for 30 minutes. Next, DENV1-4 antigen mix is added to the wells, and the plates are again incubated, followed by washing. Serum samples, diluted according to the patient's DENV history as either a single 1:10 dilution (if the child was DENV-negative in the previous years) or four 10-fold dilutions (if the child is DENV-immune), are then added and incubated. A negative control (normal human serum) and a positive control (serum with high DENV-specific antibody titer) are also included on the plate. Following another incubation period and washing, a horse-radish peroxidase (HRP)-conjugated secondary antibody is added. After a final incubation and wash, the colorimetric peroxidase substrate TMB is introduced, and the plates are protected from light during a room temperature incubation of 30 minutes. The reaction is stopped with sulfuric acid, and absorbance is read at 450/630nm. The percentage of inhibition for each sample is determined relative to the negative control's absorbance, considering 100% as the mean negative control absorbance.

Seroconversion is identified when absorbance at a 1:10 dilution is less than 50% in the first year but equal to or more than 50% in the subsequent year. If percent inhibition is under 50% at the 1:10 dilution, the titer is reported as <1:10. For those who have seroconverted previously, titration of serial dilutions

enables estimation of the iELISA titer using the Reed-Muench method[16], which accounts for the dilution factor and the percent inhibition before and after the 50% inhibition threshold.

Typing of primary inapparent infection based on EDIII-MMBA

During the evaluation phase, the serotype was assigned based on the highest MFI value observed among the set of EDIII antigens. In order to define more stringent rules to serotype an inapparent infection in the larger cohort set, we analyzed the pattern of cross-reactivity observed in the evaluation set using the MFI values of the EDIII set at a serum dilution of 1:800, chosen because this dilution was the common dilution outside of the prozone for all samples in the evaluation set. Specifically, the MFI values for EDIII-DENV1, EDIII-DENV2, EDIII-DENV3, EDIII-DENV4, and EDIII-ZIKV were expressed as the relative proportion of the highest MFI observed. We generated a heatmap (example shown in Fig. 2) with hierarchical clustering by Wald's method, which resulted in 5 distinct clusters separating DENV1-4 and ZIKV with accuracy, also providing visual support for analyzing cross-reactivity patterns (examples shown in Fig. 2). Based on this analysis, we established empirical cut-off values for each serotype based on the relative MFI observed in the evaluation set by comparing the relative proportion of the second-highest MFI to the highest MFI observed. Specifically, the cut-off values applied derived from the evaluation set applied to the cohort analysis were as follows: second highest MFI observed less than 70% of the DENV1 signal in DENV1 primary infection, less than 30% of the DENV2 signal in DENV2 primary infection, less than 60% of the DENV3 signal in DENV3 primary infection, less than 40% of the DENV4 signal in DENV4 primary infection, and less than 20% of the ZIKV signal in ZIKV primary infection.

Next, the EDIII-MMBA was applied to the larger set of the PDCS, and serotyping was performed according to two criteria: (A) the serotype was assigned based on the highest MFI value observed among a set of EDIII antigens, and (B) the validity of the serotyping was assessed according to the empirical cutoff mentioned above. Serotyping was considered valid when the second highest relative MFI value was below the empirical cutoff value of the respective maximum MFI. For example, if the maximum MFI indicated a DENV1 serotype, the serotyping was valid if the second highest MFI was below 70%; it was invalid if the second highest MFI was above 70%.

Evaluation of the EDIII-MMBA

The evaluation of the EDIII-MMBA for serotyping primary inapparent infection involved two phases, both using samples from the PDCS: (1) evaluation using samples from participants with symptomatic primary DENV infections, and (2) evaluation using samples from participants with primary inapparent DENV infections. Samples were selected by convenience, which were readily available at UC Berkeley with sufficient volume, infection history data showing naïve DENV status by DENV iELISA prior to the infection event, RT-PCR results in the case of symptomatic DENV infections and neutralization data in the case of inapparent infections. The EDIII MMBA data was generated after sample selection, and samples were processed blindly. The results obtained were compared against the gold standard serotyping results conducted by an independent researcher. Of note, DENV4 circulated at epidemic levels in 2022-2023, right after our study ended. We then had the opportunity to include several DENV4 samples from this epidemic to evaluate our EDIII-MMBA assay against DENV4 primary infection serotyped by RT-PCR.

Phase I

Serum samples were selected from 70 participants with symptomatic primary DENV infections (DENV1=16, DENV2=13, DENV3=20, DENV4=6, and ZIKV=15), confirmed by gold-standard realtime RT-PCR.

Phase II
Serum samples were selected from 86 participants with primary inapparent DENV infections (DENV1=38, DENV2=19, DENV3=19, and ZIKV=10), confirmed by gold-standard antibody focus reduction neutralization test."

Performance evaluation

The accuracy of the EDIII-MMBA for serotype identification in primary DENV infections was evaluated by comparing it to gold standards: RT-PCR and FRNT assays. Sensitivity, specificity, and accuracy, the latter calculated as the sum of the true positives and true negatives divided by total number of samples evaluated, were calculated for each DENV serotype and Zika virus (ZIKV) across all test combinations. We calculated 95% confidence intervals using the exact binomial method for all the performance measures.

Covariate definition and relationship between variables

Covariates in our study include: DENV serotype, epidemic year, neighborhood and household location.

DENV serotype: DENV serotype determined by RT-PCR or EDIII-MMBA in symptomatic primary DENV infections or inapparent primary DENV infections, respectively. This exposure variable was analyzed as a categorical variable.

Epidemic year: The epidemic year starts between February-April (in July before 2011), corresponding to the start of the annual sampling, and ends at the beginning of the next annual sampling. During the annual sampling this period, the season is dry and DENV circulation is low. Cases usually peak between September and October during the wet season. This covariate was analyzed as a categorical variable.

Neighborhood: Neighborhood was defined by political administration by the Nicaraguan Census where participants live. This covariate was analyzed as a categorical variable.

Variable relationship: A causal framework for the main endpoint analysis (e.g. ratio of symptomatic versus inapparent and severe versus non-severe DENV infections by serotype) was created using Directed Acyclic Graph (DAG). Yellow shade shows exposure variables while pink shade shows the outcome variable. White boxes show adjusted/controlled variables. Black lines show closed paths, and the green lines show unbiased open path. The dashed line shows the presumptive causal relationship. The analyses were restricted to individuals with naïve status and adjusted by year. Age was adjusted in both the imputation models and main endpoint models and are shown as sensitivity analyses.

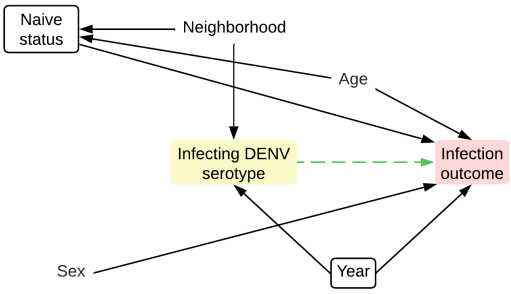

Sensitivity analyses

We performed several sensitivity analyses on our main endpoint models, including: (1) logistic regression analysis on the experimentally serotyped data, excluding missing data; (2) addition of the "no EDIII-Abs detected" category into the polytomous regression to address the potential limitation of the EDIII-MMBA to serotype primary infections detected by iELISA; (3) performing imputations with various frameworks, (3) including the non-parametric models (not assuming a specific form for the relationship between

predictors and the response) such as regression trees, which partition the data into subsets based on feature values, creating a tree-like model for predictions being easy to interpret but prone to overfitting; (4) random forest, which is an ensemble method that combines the predictions from multiple decision trees to make more accurate and stable estimations being more robust to overfitting due to the averaging of multiple trees; (5) the semi-parametric predictive mean matching framework, which retains the original distribution of the data assuming that the distribution of the missing data is similar to the observed data; (6) adjusting the endpoint models not only by year but also by age and biological sex (no genetic confirmation); (7) changing the covariates of our imputation models to include only year; (8) adding year with an interaction term with region (neighborhoods classified by northeast, northwest, southeast, and southwest); (9) incorporating year with an interaction term with region plus age; (10) ignoring DENV4 cases and imputing only DENV1-3 cases; (11) restricting our analyses from 2004 to 2015 to only cover the pre-ZIKV era; (12) removing the 76 samples from the EDIII-MMBA validation, which were selected by convenience sampling; and (13) excluding the years with counts of primary infections that are below 30 (2014, 2016-18, 2020-21). All imputation models to address missing data were stratified by inapparent and symptomatic infections and all main endpoint models were adjusted by year.

We tested multiple frameworks to ensure that our analyses were not compromised by incorrect or overly restrictive assumptions or by the selection of models susceptible to bias or variance. For instance, simpler models such as polytomous regression often exhibit high bias with low variance, whereas more complex models, like random forests, typically exhibit low bias yet high variance. Finally, we assessed models based on a trade-off between interpretability and accuracy. While polytomous regression models are highly interpretable (the optimal), random forests, despite their accuracy, tend to be less so.

Evaluation of the final imputation models

We first examined trace plots to assess the convergence of our final imputation models, which adjust for year and neighborhood, constructed using 1000 simulations and 10 iterations. To further scrutinize visually the convergence and potential trends within the models on a more granular level, we performed additional runs with reduced simulations (10 simulations) across various numbers of iterations (10 and 30) to ensure stability across iterations. Additionally, we compared the model outputs to experimentally serotyped data, focusing on metrics such as overall accuracy, kappa statistics, and serotype-specific sensitivity and specificity. Lastly, we assured that the models were effective in estimating the distribution of the number of individuals by serotype and year, which was essential as our primary goal was to infer statistical parameters rather than individual imputations.

Supplementary figures and tables

Table S1. Count of naïve participants and primary DENV infections per year

	Naive	Infection		Inapparent		Symptomatic					
								DENV1	DENV2	DENV3	Serology[1]
	n	n	%	n	%	n	%	n	n	n	n
2004	1289	124	9·62	116	93·55	8	6·45	6	0	0	2
2005	1328	150	11·3	129	86	21	14·00	10	10	0	1
2006	1406	79	5·62	74	93·67	5	6·33	1	4	0	0
2007	1511	123	8·14	99	80·49	24	19·51	1	22	0	1
2008	1713	87	5·08	70	80·46	17	19·54	0	2	14	1
2009	1595	202	12·66	137	67·82	65	32·18	8	1	53	3
2010	1433	123	8·58	80	65·04	43	34·96	0	5	32	6
2011	1624	59	3·63	44	74·58	15	25·42	3	0	10	2
2012	1861	124	6·66	75	60·48	49	39·52	44	1	1	3
2013	2082	65	3·12	45	69·23	20	30·77	11	2	4	3
2014	2115	25	1·18	18	72	7	28·00	0	0	1	6
2015	2543	80	3·15	68	85	12	15·00	0	8	0	4
2016	2556	9	0·35	2	22·22	7	77·78	0	3	0	4
2017	1425	14	0·98	14	100	0	0·00	0	0	0	0
2018	1492	5	0·34	3	60	2	40·00	0	1	0	1
2019	1356	287	21·17	203	70·73	84	29·27	0	47	0	37
2020	1459	15	1·03	12	80	3	20·00	0	1	0	2
2021	1561	20	1·28	20	100	0	0·00	0	0	0	0

[1] Positive according to serological assays (MAC-ELISA, or iELISA), with inconclusive RT-PCR results.

Table S2. Breakdown of serotyped and non-serotyped inapparent and symptomatic primary DENV infections

	Inapparent				Symptomatic		
Cohort year	Overall n (%)	Screened n (%)	Typed[1] n (%)	Non-screened[2] n (%)	Overall n (%)	Typed[1] n (%)	Non-typed[2] n (%)
2004	116 (100)	46 (40)	40 (34)	76 (66)	8 (100%)	6 (75%)	2 (25%)
2005	129 (100)	50 (39)	39 (30)	90 (70)	21 (100%)	20 (95%)	1 (4·8%)
2006	74 (100)	30 (41)	13 (18)	61 (82)	5 (100%)	5 (100%)	0 (0%)
2007	99 (100)	38 (38)	23 (23)	76 (77)	24 (100%)	23 (96%)	1 (4·2%)
2008	70 (100)	27 (39)	14 (20)	56 (80)	17 (100%)	16 (94%)	1 (5·9%)
2009	137 (100)	53 (39)	43 (31)	94 (69)	65 (100%)	62 (95%)	3 (4·6%)
2010	80 (100)	34 (43)	32 (40)	48 (60)	43 (100%)	39 (91%)	4 (9·3%)
2011	44 (100)	31 (70)	31 (70)	13 (30)	15 (100%)	13 (87%)	2 (13%)
2012	75 (100)	48 (64)	48 (64)	27 (36)	49 (100%)	48 (98%)	1 (2·0%)
2013	45 (100)	19 (42)	19 (42)	26 (58)	20 (100%)	18 (90%)	2 (10%)
2014	18 (100)	17 (94)	5 (28)	13 (72)	7 (100%)	1 (14%)	6 (86%)
2015	68 (100)	30 (44)	30 (44)	38 (56)	12 (100%)	8 (67%)	4 (33%)
2016	2 (100)	2 (100)	1 (50)	1 (50)	7 (100%)	3 (43%)	4 (57%)
2017	14 (100)	12 (86)	4 (29)	10 (71)	0 (0%)	0 (0%)	0 (0%)
2018	3 (100)	3 (100)	0 (0)	3 (100)	2 (100%)	1 (50%)	1 (50%)
2019	203 (100)	69 (34)	68 (33)	135 (67)	84 (100%)	47 (56%)	37 (44%)
2020	12 (100)	11 (92)	8 (67)	4 (33)	3 (100%)	1 (33%)	2 (67%)
2021	20 (100)	19 (95)	7 (35)	13 (65)	0 (0%)	0 (0%)	0 (0%)

[1]Typed: DENV serotype determined by RT-PCR (symptomatic infection) or EDIII-MMBA (inapparent infection).
[2]Missing serotype information. Stochastic imputation was applied to deal with missingness.

Table S3. Imputation model performance on inapparent infections based on the experimentally serotyped data

Serotype	Prevalence	Accuracy	Sensitivity	Specificity	Overall accuracy	Overall kappa
DENV1	0·29	0·79	0·65	0·93	0·78 (0·74-0·82)	0·66
DENV2	0·47	0·84	0·85	0·83		
DENV3	0·22	0·89	0·86	0·91		
DENV4	0·02	0·66	0·33	0·99		

Table S4. Imputation model performance on symptomatic infections based on the experimentally serotyped data

Serotype	Prevalence	Accuracy	Sensitivity	Specificity	Overall accuracy	Overall kappa
DENV1	0·28	0·87	0·77	0·97	0·89 (0·85-0·92)	0·83
DENV2	0·35	0·94	0·92	0·96		
DENV3	0·37	0·93	0·96	0·90		

Table S5. Actual vs predicted inapparent DENV infections based only on the experimentally serotyped data

		DENV1		DENV2		DENV3		DENV4	
**Year**	**n**	**Actual**	**Predicted**	**Actual**	**Predicted**	**Actual**	**Predicted**	**Actual**	**Predicted**
2004	40	17	19	17	18	1	0	4	4
2005	39	14	1	37	22	2	1	1	0
2006	13	3	0	13	10	0	0	0	0
2007	23	5	1	22	16	2	0	0	0
2008	14	4	7	2	4	5	5	1	0
2009	43	10	0	1	8	25	42	0	0
2010	32	0	0	2	6	26	30	0	0
2011	31	3	0	3	4	23	28	1	0
2012	48	45	48	0	1	2	0	0	0
2013	19	15	19	0	3	1	0	0	0
2014	5	1	0	1	1	2	2	1	2
2015	30	0	0	30	27	2	0	1	0
2016	1	0	0	1	1	0	0	0	0
2017	4	2	3	1	1	1	0	0	0
2019	68	0	0	68	68	0	0	0	0
2020	8	1	0	8	7	0	0	0	0
2021	7	4	5	2	3	0	0	0	0

Table S6. Actual vs predicted symptomatic DENV infections based only on the experimentally serotyped data

		DENV1		DENV2		DENV3	
Year	n	Actual	Predicted	Actual	Predicted	Actual	Predicted
2004	6	6	3	3	0	0	0
2005	20	10	8	12	10	0	0
2006	5	1	0	5	4	0	0
2007	23	1	0	23	22	0	0
2008	16	0	0	1	2	14	15
2009	62	8	3	0	1	53	59
2010	39	1	0	3	6	32	36
2011	13	3	0	0	0	10	13
2012	48	46	48	0	1	1	0
2013	18	11	12	0	2	5	6
2014	1	0	0	0	0	1	1
2015	8	0	0	8	8	0	0
2016	3	0	0	3	3	0	0
2018	1	0	0	1	1	0	0
2019	47	0	0	47	47	0	0
2020	1	0	0	1	1	0	0

Table S7. Significance of fisher tests

Cohort year	Imputed dataset		Typed dataset	
	p.value[1]	p.star[2]	p.value	p.star
2004	0·0105	*	0·0665	ns
2005	0·7686	ns	0·6877	ns
2006	1·0000	ns	1·0000	ns
2007	0·0205	*	0·0535	·
2008	0·0025	**	0·0065	**
2009	0·0005	***	0·0010	***
2010	0·2229	ns	0·8646	ns
2011	0·1959	ns	0·4423	ns
2012	1·0000	ns	1·0000	ns
2013	0·1894	ns	0·2179	ns
2014	0·1299	ns	1·0000	ns
2015	0·2639	ns	1·0000	ns
2016	1·0000	ns	-	-
2018	1·0000	ns	-	-
2019	0·0755	ns	-	-
2020	0·6082	ns	1·0000	ns

[1] p-value as determined by fisher test.

[2] *, p<0·05; **, p<0·01; ***, p<0·005.

Table S8. Overall incidence of primary DENV infections by serotype.

Serotype	Incidence*	Upper 95% CI	Lower 95% CI
DENV1	2·70	3·03	2·40
DENV2	3·71	4·01	3·42
DENV3	2·66	2·98	2·37

**This analysis excludes years when no symptomatic infections were reported. This is the result of intercept-only models. Incidence was calculated as the number of cases per serotype over the total population at risk (DENV-naïve individuals) times 100.*

Table S9. Risk of dengue infections and percent symptomatic given primary infection by dengue serotype over time, based on model predictions across imputations excluding years with low symptomatic circulation (n < 10).

	DENV1		DENV2		DENV3	
Year	Incidence[1]	Percent symptomatic	Incidence[1]	Percent symptomatic	Incidence[1]	Percent symptomatic
2004	4 (3-5)	15 (8-28)	-	-	-	-
2005	4 (3-5)	19 (10-32)	6 (5-7)	12 (7-22)	-	-
2006	1 (1-2)	6 (1-35)	4 (3-5)	6 (2-16)	-	-
2007	1 (1-2)	4 (1-26)	5 (4-7)	26 (18-36)	-	-
2008	-	-	1 (1-2)	8 (2-29)	2 (2-3)	38 (23-55)
2009	2 (1-3)	22 (11-39)	2 (1-3)	3 (0-20)	7 (6-9)	42 (34-51)
2010	-	-	2 (1-3)	25 (11-46)	6 (5-7)	37 (28-48)
2011	-	-	-	-	3 (2-4)	25 (15-40)
2012	6 (5-7)	40 (32-49)	-	-	-	-
2013	2 (2-3)	26 (16-41)	-	-	-	-
2014	-	-	-	-	1 (0-1)	44 (22-69)
2015	-	-	3 (2-4)	16 (9-27)	-	-
2016	-	-	-	-	-	-
2017	-	-	-	-	-	-
2018	-	-	-	-	-	-
2019	-	-	17 (15-19)	29 (24-35)	-	-
2020	-	-	1 (0-1)	24 (8-54)	-	-
2021	-	-	-	-	-	-

[1]Incidence per 100 naive individuals

Table S10. Overall incidence of primary DENV infections by serotype accounting for clustering.

	Intercept			Random effect
Serotype	estimate*	CI95U	CI95L	Participant ID - SD**
DENV1	2.7	3.03	2.4	0.00000226
DENV2	3.82	4.15	3.52	0.73
DENV3	2.72	3.06	2.42	0.752

*This analysis excludes years when no symptomatic infections were reported. This is the result of intercept-only models. Incidence was calculated as the number of cases per serotype over the total population at risk (DENV-naïve individuals) times 100.
** Mean standard deviation of participant ID as cluster variable across imputations

Table S11. Risk of dengue infections and percent symptomatic given primary infection by dengue serotype over time, based on model predictions across imputations excluding years with low symptomatic circulation (n < 10) accounting for clustering.

	Variable	DENV1 incidence*	DENV2 incidence*	DENV3 incidence*
Fixed effects	2004	4 (3-5)	-	-
	2005	4 (3-5)	6 (5-7)	-
	2006	1 (1-2)	4 (3-5)	-
	2007	1 (1-2)	5 (4-7)	-
	2008	-	1 (1-2)	2 (2-3)
	2009	2 (1-3)	2 (1-3)	7 (6-9)
	2010	-	2 (1-3)	6 (5-8)
	2011	-	-	3 (2-4)
	2012	6 (5-7)	-	-
	2013	2 (2-3)	-	-
	2014	-	-	1 (0-1)
	2015	-	3 (2-4)	-
	2016	-	-	-
	2017	-	-	-
	2019	-	17 (15-19)	-
	2020	-	1 (0-1)	-
	2021	-	-	-
Random effect	Participant ID - SD**	0.00000183	0.00000349	0.00000223

* Incidence per 100 individuals
** Mean standard deviation of participant ID as cluster variable across imputations

Table S12. Odds ratios of symptomatic primary infection given DENV1 infection over time

Year	Pooled OR	Std error	Low 95% CI	High 95% CI	P-value
2004	0.26	0.44	0.11	0.62	0.002
2005	0.34	0.41	0.15	0.76	0.009
2006	0.1	1.07	0.01	0.84	0.034
2007	0.07	1.05	0.01	0.54	0.011
2009	0.42	0.46	0.17	1.04	0.061
2012	ref	ref	ref	ref	ref
2013	0.54	0.39	0.25	1.16	0.11

Table S13. Odds ratios of symptomatic primary infection given DENV2 infection over time

Year	Pooled	Std error	Low 95% CI	High 95% CI	P-value
2005	0.34	0.36	0.17	0.7	0.003
2006	0.16	0.54	0.06	0.47	< 0.001
2007	0.84	0.28	0.48	1.45	0.53
2008	0.22	0.76	0.05	0.99	0.049
2009	0.08	01.03	0.01	0.63	0.016
2010	0.79	0.51	0.29	2.14	0.65
2015	0.47	0.34	0.24	0.92	0.028
2019	ref	ref	ref	ref	ref
2020	0.76	0.68	0.2	2.89	0.68

Table S14. Odds ratios of symptomatic primary infection given DENV3 infection over time

Year	Pooled	Std error	Low 95% CI	High 95% CI	P-value
2008	0.84	0.4	0.38	1.86	0.67
2009	ref	ref	ref	ref	ref
2010	0.81	0.29	0.46	1.42	0.46
2011	0.47	0.39	0.21	01.01	0.054
2014	01.08	0.57	0.35	3.31	0.89

Table S15. Sensitivity analyses

Sensitivity analysis	Serotype	Outcome	Event	Infected	Percent	Pooled OR (95% CI)	P-value
No imputation - experimentally typed data	DENV1	Symptomatic given infection	87	211	41·23	Ref.	Ref.
	DENV2		108	308	35·06	0·78 (0·44 to 1·39)	0·40
	DENV3		116	208	55·77	1·83 (1·01 to 3·34)	0·046
	DENV1	Severe given infection	10	211	4·74	Ref	Ref
	DENV2		9	308	2·92	1·12 (0·24 to 4·93)	0·89
	DENV3		26	208	12·5	5·61 (1·69 to 21·17)	0·0072
Polytomous regression framework (imputing DENV-infected with no EDIII)	DENV1	Symptomatic given infection	94	337	27·89	Ref	Ref
	DENV2		157	663	23·68	0·83 (0·50 to 1·36)	0·455
	DENV3		131	318	41·19	1·81 (1·08 to 3·05)	0·025
	DENV1	Severe given infection	10	337	2·97	Ref	Ref
	DENV2		11	663	1·66	1·19 (0·29 to 4·90)	0·808
	DENV3		27	318	8·49	5·60 (1·69 to 18·52)	0·005
Classification trees imputation framework	DENV1	Symptomatic given infection	96	418	22·97	Ref	Ref
	DENV2		159	781	20·36	0·93 (0·57 to 1·54)	0·787
	DENV3		126	366	34·43	1·97 (1·18 to 3·28)	0·01
	DENV1	Severe given infection	10	418	2·39	Ref	Ref
	DENV2		11	781	1·41	1·38 (0·34 to 5·56)	0·651
	DENV3		27	366	7·38	6·39 (1·96 to 20·88)	0·002
Random forest framework	DENV1	Symptomatic given infection	96	426	22·54	Ref	Ref
	DENV2		155	768	20·18	0·91 (0·56 to 1·46)	0·682
	DENV3		130	368	35·33	2·18 (1·32 to 3·59)	0·002
	DENV1	Severe given infection	10	426	2·35	Ref	Ref
	DENV2		11	768	1·43	1·31 (0·35 to 4·84)	0·687
	DENV3		27	368	7·34	5·94 (1·91 to 18·50)	0·002
Predictive mean matching imputation framework	DENV1	Symptomatic given infection	97	413	23·49	Ref	Ref
	DENV2		151	774	19·51	0·91 (0·54 to 1·55)	0·729
	DENV3		135	377	35·81	2·14 (1·10 to 4·17)	0·025
	DENV1	Severe given infection	10	413	2·42	Ref	Ref
	DENV2		11	774	1·42	1·37 (0·38 to 4·89)	0·63
	DENV3		27	377	7·16	6·35 (1·86 to 21·66)	0·003
Polytomous regression (outcome models adjusted by age and sex)	DENV1	Symptomatic given infection	94	402	23·38	Ref	Ref
	DENV2		157	782	20·08	0·78 (0·46 to 1·32)	0·357
	DENV3		131	363	36·09	2·36 (1·37 to 4·09)	0·002
	DENV1	Severe given infection	10	402	2·49	Ref	Ref
	DENV2		11	782	1·41	1·20 (0·23 to 6·37)	0·83
	DENV3		27	363	7·44	10·02 (2·19 to 45·83)	0·003
Polytomous regression framework (serotype ~ year)	DENV1	Symptomatic given infection	93	418	22·25	Ref	Ref
	DENV2		158	761	20·76	0·98 (0·60 to 1·60)	0·933
	DENV3		131	377	34·75	2·09 (1·25 to 3·50)	0·005
	DENV1	Severe given infection	10	418	2·39	Ref	Ref
	DENV2		11	761	1·45	1·39 (0·35 to 5·59)	0·639
	DENV3		27	377	7·16	6·30 (1·93 to 20·56)	0·002

Sensitivity analysis	Serotype	Outcome	Event	Infected	Percent	Pooled OR (95% CI)	P-value
Polytomous regression framework (serotype ~ region*year)	DENV1	Symptomatic given infection	93	415	22·41	Ref	Ref
	DENV2		158	764	20·68	0·90 (0·55 to 1·48)	0·688
	DENV3		131	375	34·93	2·10 (1·25 to 3·51)	0·005
	DENV1	Severe given infection	10	415	2·41	Ref	Ref
	DENV2		11	764	1·44	1·40 (0·35 to 5·59)	0·634
	DENV3		27	375	7·2	6·28 (1·93 to 20·41)	0·002
Polytomous regression framework (serotype ~ region*year + age)	DENV1	Symptomatic given infection	92	403	22·83	Ref	Ref
	DENV2		154	722	21·33	1·01 (0·62 to 1·66)	0·955
	DENV3		134	368	36·41	2·40 (1·45 to 3·97)	<0·001
	DENV1	Severe given infection	10	403	2·48	Ref	Ref
	DENV2		11	722	1·52	1·43 (0·37 to 5·47)	0·603
	DENV3		27	368	7·34	6·34 (2·06 to 19·55)	0·001
Ignoring DENV4 infections	DENV1	Symptomatic given infected	94	419	22·43	Ref	Ref
	DENV2		157	797	19·7	0·87 (0·54 to 1·41)	0·58
	DENV3		131	375	34·93	2·09 (1·27 to 3·43)	0·004
	DENV1	Severe given infected	10	419	2·39	Ref	Ref
	DENV2		11	797	1·38	1·22 (0·31 to 4·79)	0·775
	DENV3		27	375	7.2	6·45 (1·97 to 21·15)	0·002
Constraining study period to 2004-2016 (Prior to ZIKV emergence)	DENV1	Symptomatic given infected	93	379	24·54	Ref	Ref
	DENV2		63	461	13·67	0·77 (0·47 to 1·27)	0·312
	DENV3		131	360	36·39	2·12 (1·26 to 3·56)	0·005
	DENV1	Severe given infected	10	379	2·64	Ref	Ref
	DENV2		5	461	1·08	0·99 (0·24 to 4·14)	0·987
	DENV3		27	360	7·50	6·41 (1·93 to 21·35)	0·002
Removing the 76 samples from the EDIII-MMBA evaluation set	DENV1	Symptomatic given infected	94	374	25·13	Ref	Ref
	DENV2		157	746	21·05	0·83 (0·50 to 1·38)	0·482
	DENV3		131	346	37·86	2·12 (1·25 to 3·59)	0·006
	DENV1	Severe given infected	10	374	2·67	Ref	Ref
	DENV2		11	746	1·47	1·16 (0·29 to 4·73)	0·833
	DENV3		27	346	7·80	6·33 (1·83 to 21·83)	0·004
Removing years with count of primary infections <30 (2014, 2016-2018, 2020-2021)	DENV1	Symptomatic given infected	93	379	24·54	Ref	Ref
	DENV2		146	738	19·78	0·77 (0·47 to 1·27)	0·302
	DENV3		124	343	36·15	2·03 (1·21 to 3·41)	0·008
	DENV1	Severe given infected	10	379	2·64	Ref	Ref
	DENV2		10	738	1·36	1·04 (0·25 to 4·32)	0·961
	DENV3		27	343	7·87	6·54 (1·96 to 21·90)	0·002

Table S16. Outcome of primary infections

		Symptomatic			
**Serotype**	**Inapparent**	**Overall**	**DwoWS**	**DwWS**	**SD**
**DENV1**	76.8% (72.1-80.8%)	23.2% (19.2-27.8%)	11.2% (8.4-14.8%)	9.5% (7.0-12.9%)	2.5% (1.3-4.6%)
**DENV2**	79.9% (76.8-82.6%)	20.1% (17.4-23.2%)	14.1% (11.8-16.8%)	4.6% (3.3-6.3%)	1.4% (0.8-2.6%)
**DENV3**	63.9% (58.6-68.9%)	36.1% (31.1-41.4%)	16.4% (12.8-20.7%)	12.3% (9.3-16.2%)	7.4% (5.1-10.6%)

Table S17. Genotype of Nicaraguan DENV serotypes.

Serotype	Genotype
DENV1	Genotype V (American/East African and Asian)
DENV2	Genotype IIIb (Southeast Asian-American)
DENV3	Genotype III lineage C2 (Indian-subcontinent)

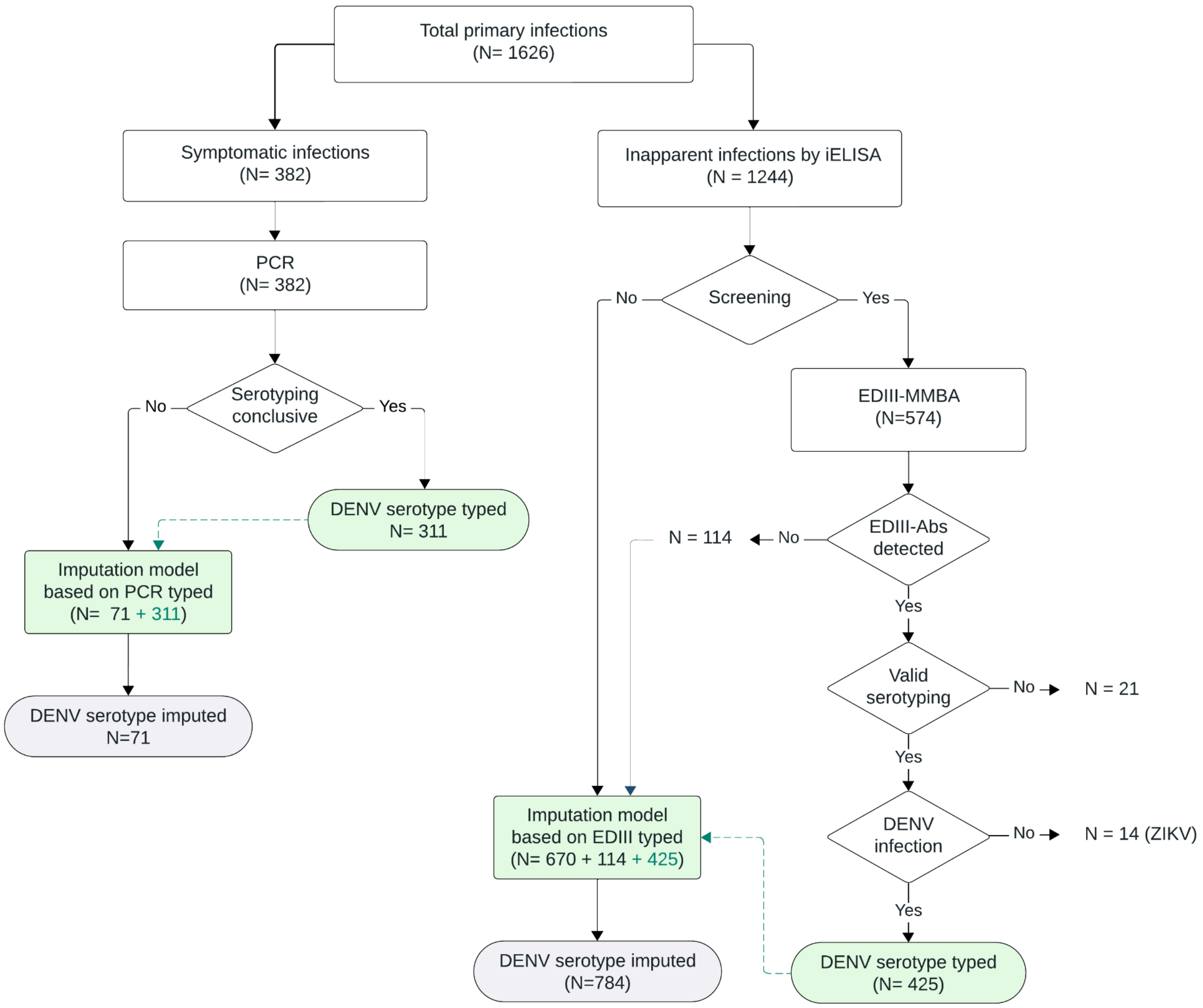

Figure S1. Flowchart of the screening and serotyping process to study the epidemiology of primary DENV infections in the PDCS

Serotyping inconclusive refers to participants with primary symptomatic infection via MAC-ELISA and/or iELISA, with no serotyping data by RT-PCR due to negative, unclear results or low viremia. Valid serotyping refers to participants for whom conclusive serotyping was performed via EDIII-MMBA according to the rules detailed in the Supplementary Appendix, section “Typing of primary inapparent infection based on EDIII-MMBA”.

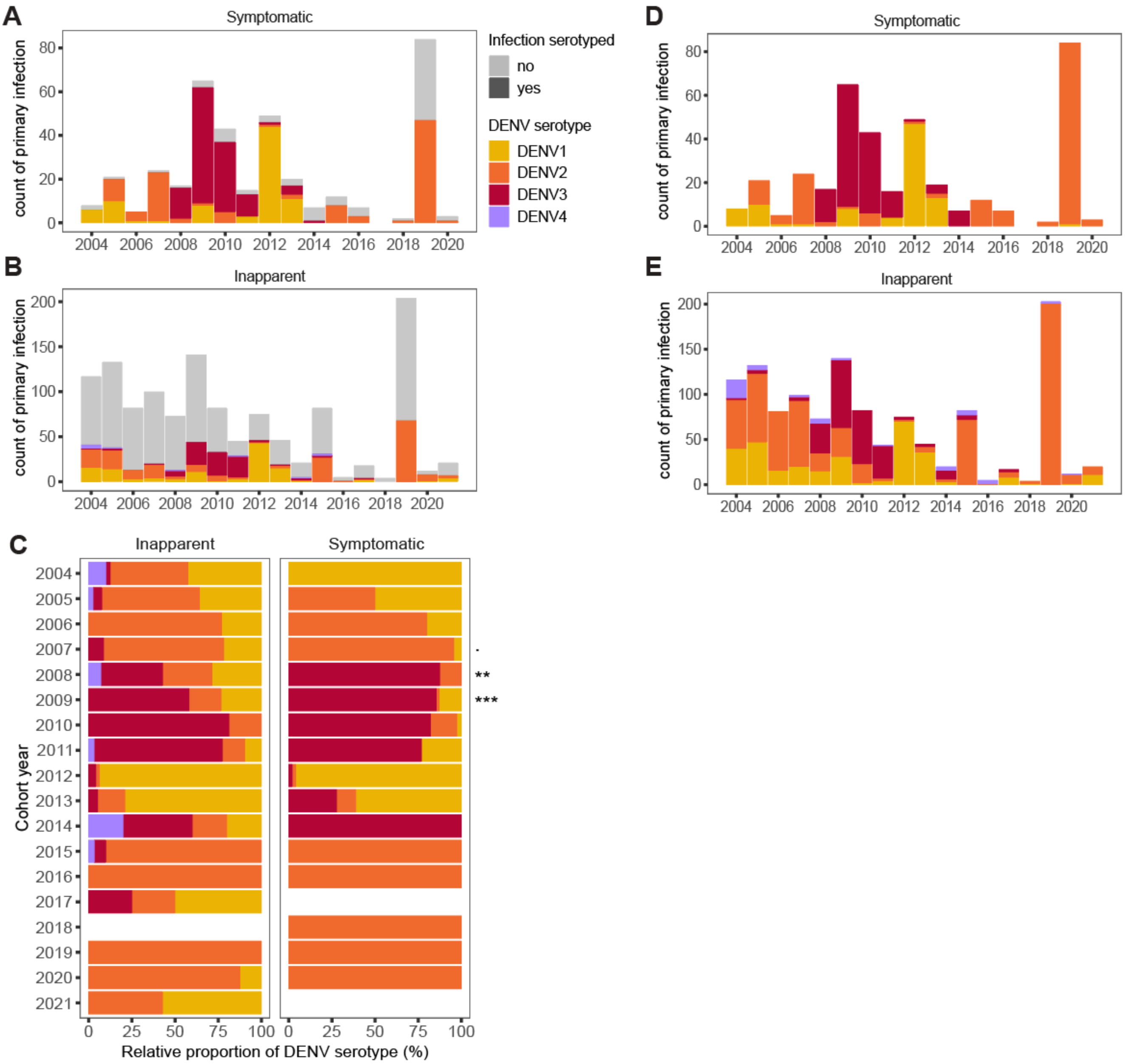

Figure S2. Distribution of serotyped, non-serotyped, and imputed symptomatic and inapparent primary DENV from 2004 to 2021 in the PDCS

(A) Yearly counts of symptomatic primary DENV infections by DENV serotype as serotyped by rRT-PCR. **(B)** Yearly counts of inapparent primary DENV infections by DENV serotype as serotyped by EDIII-MMBA. **(C)** Relative distribution of DENV serotypes circulating yearly in inapparent and symptomatic primary DENV infections as serotyped by rRT-PCR and EDIII-MMBA. **(D)** Pooled yearly counts of symptomatic primary DENV infections by DENV serotype across multiple imputations **(E)** Pooled yearly counts of inapparent primary DENV infections by DENV serotype across multiple imputations. p-value, p=0·05; **, p<0·01; ***, p<0·005.

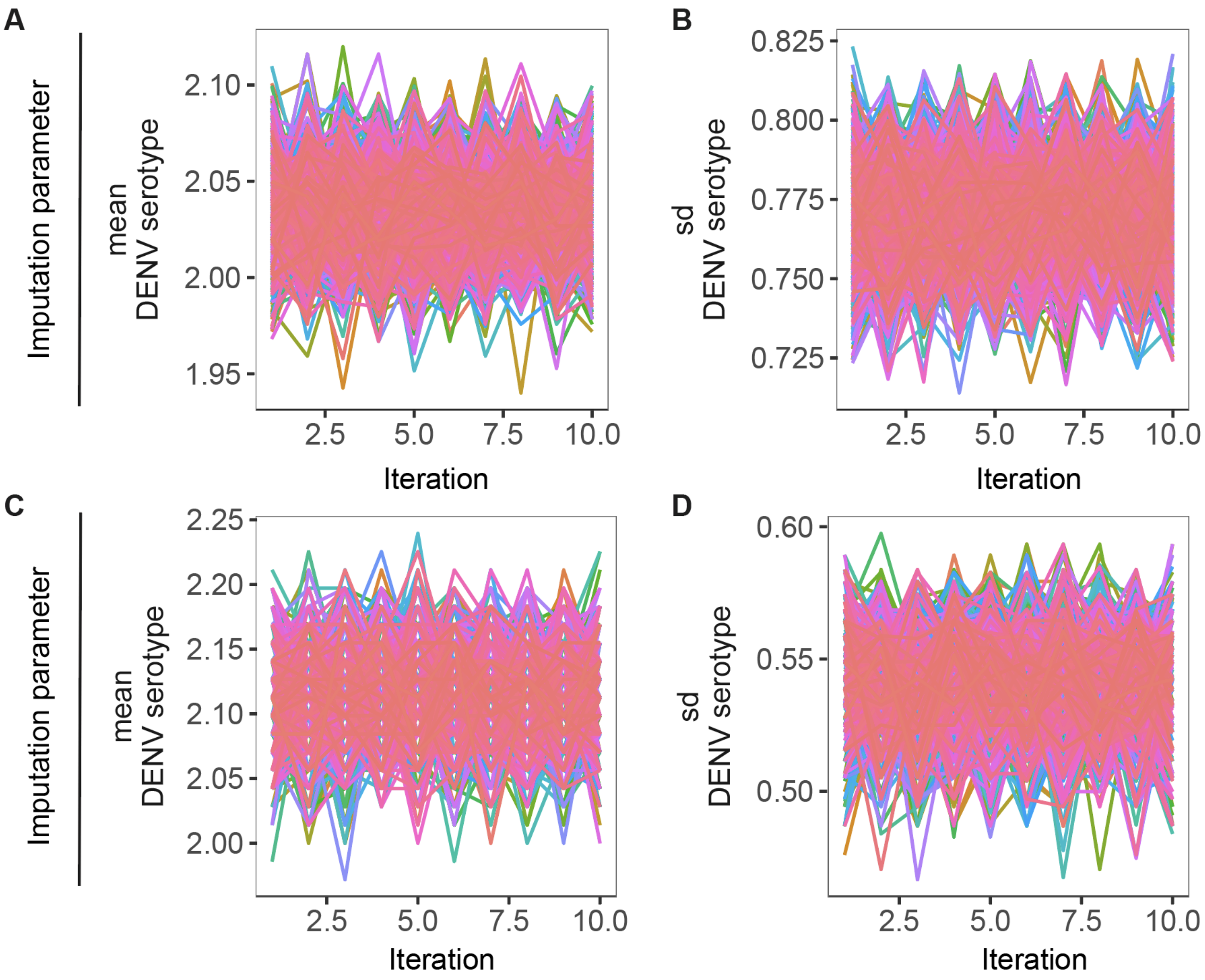

Figure S3. Trace plots for the final imputation models

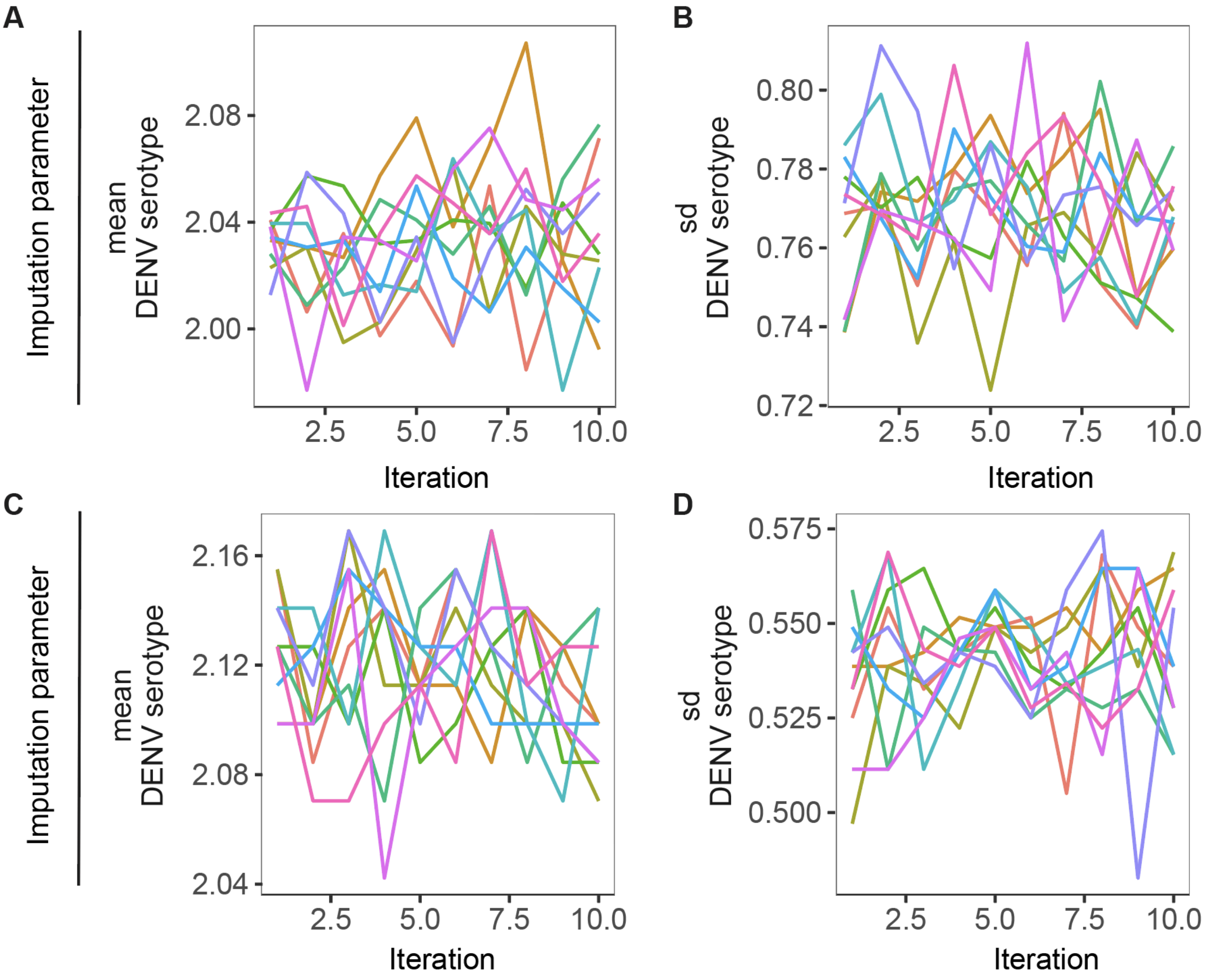

Figure S4. Trace plots for the final imputation framework based on only 10 simulations

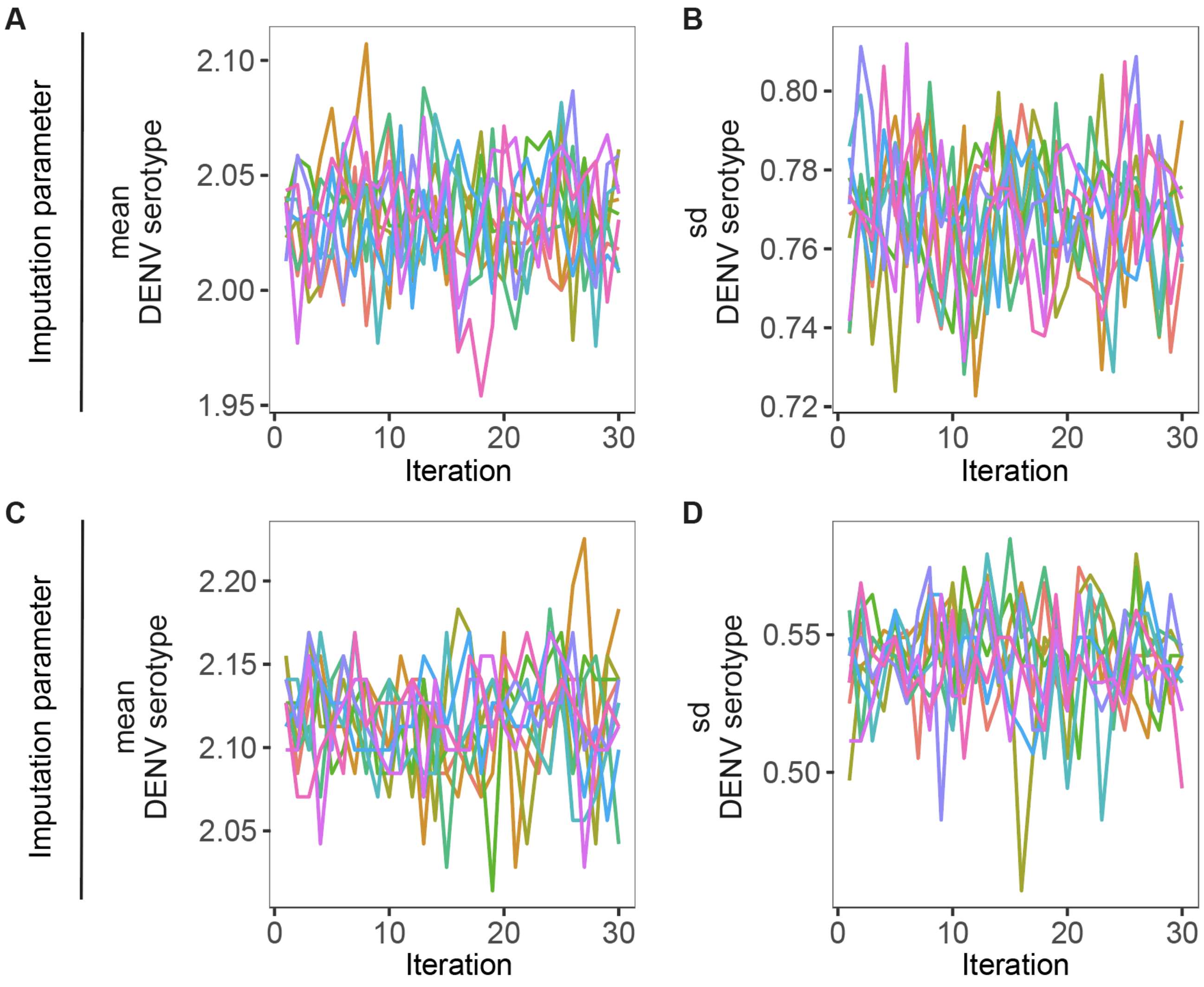

Figure S5. Trace plots for the final imputation framework based on only 10 simulations and 30 iterations

A

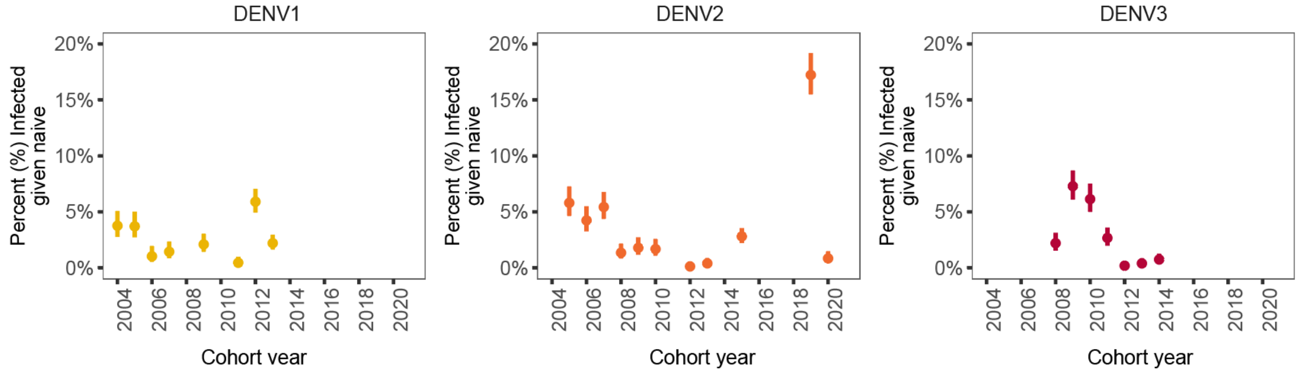

B

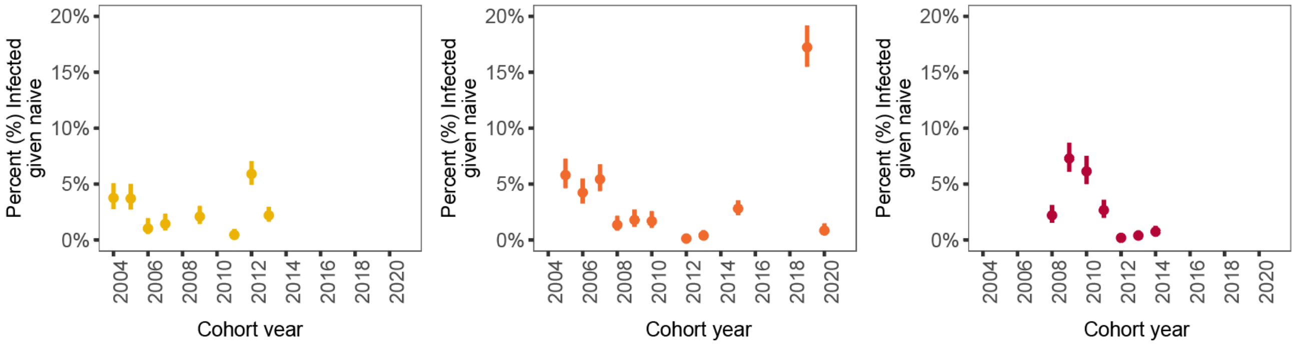

Figure S6. Temporal dynamics of overall primary DENV infections by serotype from 2004 to 2021 in the PDCS

Predictions and 95% CI of percent of primary DENV infections by infection outcome and serotype among the naive population, as indicated in left, middle, and right panels across imputations. Years without symptomatic circulation were not included. (A) not adjusted for clustering by participant identification code (B) accounting for clustering by participant identification code.

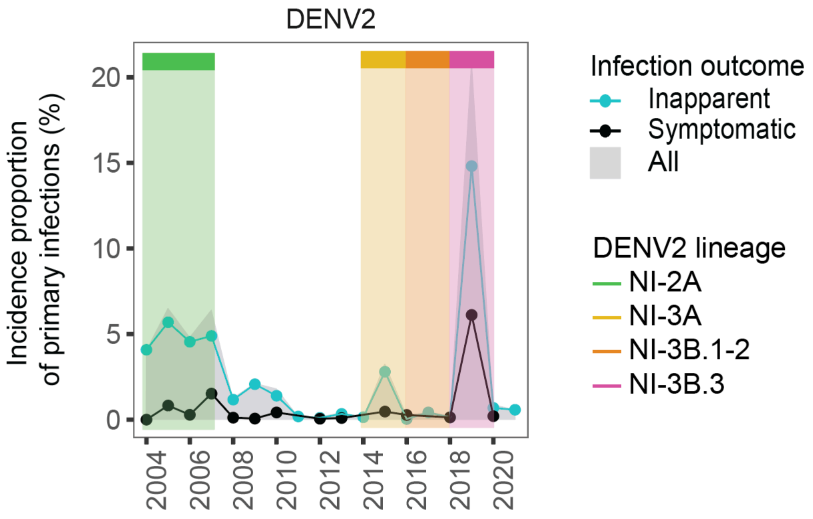

Figure S7. Temporal shift in Nicaraguan DENV2 lineages

Annual incidence proportion of primary DENV2 infections alongside the temporal evolution of DENV2 lineages in Nicaragua, mapped onto the respective years. Detailed descriptions of the Nicaraguan DENV2 lineages and pertinent information regarding amino acid mutations can be found in Thongsripong et al[17].

Supplementary Discussion

In this supplementary discussion, we would like to expand on the unusually high accuracy of the EDIII-MMBA reached to serotype primary inapparent infections in the evaluation set. We believe the exceptional performance of the assay can be attributed to 5 key parameters. (1) Study design: The point of the study was not to assess the ability of the EDIII-MMBA to detect past infections but rather to serotype prior primary infections that had already been detected by other assay(s). (2) Evaluation using samples selected based on gold standard assays: Our aim was not to determine if the EDIII-MMBA was superior to RT-PCR or neutralization assays nor to assess its ability to detect prior infections, but to confirm if it could identify the same infecting serotype as gold-standard methods. As a result, the samples for the evaluation set were pre-selected based on the capacity of gold-standard methods to call the serotype of a previously identified primary DENV infection — clear RT-PCR results for symptomatic infections or typical neutralization profiles by FRNT for primary infections. Additionally, our neutralization assays used Nicaraguan clinical isolates grown in Vero cells overexpressing furin, which increased virion maturation and reduced background neutralization by cross-reactive fusion loop antibodies.[14] Thus, post-primary infection, this setup preferentially captures neutralization mediated by type-specific responses. Given that the EDIII-MMBA relies on type-specific antibodies and that the neutralization assay also favors type-specific responses, it is possible that the samples used for EDIII-MMBA favored type-specific responses. (3) Timing of sample collection: The samples used to evaluate the EDIII-MMBA performance were collected between 6 to 12 months post-infection, like the samples used for the rest of the study. At this time-point, the difference between type-specific and cross-reactive antibody responses is maximized, enhancing the assay's accuracy, as it is far enough from the infection to reduce high cross-reactivity seen in the early convalescent phase but close enough to detect type-specific antibodies at their peak differentiation from cross-reactive antibodies. (4) Specificity of the EDIII antigen: We believe high accuracy can be achieved thanks to the nature of the EDIII antigen. EDIII is an optimal antigen due to its high proportion of type-specific epitopes and low proportion of cross-reactive epitopes relative to its size. This characteristic maximizes the capture of type-specific antibodies while minimizing the capture of cross-reactive antibodies. As shown by the heat map in Figure 2, the cross-reactivity pattern and the signature of each Nicaraguan serotype are very specific and relatively homogeneous within each serotype, creating a distinct profile that enhances serotyping accuracy. (5) Participant characteristics: Samples used in this study come from a pediatric population in an endemic country, which means that the participants likely experienced a lower number of infections compared to adults (e.g., both flaviviral and non-flaviviral), which minimizes the non-specific binding of IgG and reduces background noise. Also, no flavivirus vaccines are broadly administered in Nicaragua, which minimizes cross-reactivity and noise.

Supplementary References

1 Kuan G, Gordon A, Avilés W, *et al.* The Nicaraguan pediatric dengue cohort study: study design, methods, use of information technology, and extension to other infectious diseases. *Am J Epidemiol* 2009; **170**: 120–9.

2 Gordon A, Kuan G, Aviles W, *et al.* The Nicaraguan pediatric influenza cohort study: design, methods, use of technology, and compliance. *BMC Infect Dis* 2015; **15**: 504.

3 Zambrana JV, Bustos Carrillo F, Burger-Calderon R, *et al.* Seroprevalence, risk factor, and spatial analyses of Zika virus infection after the 2016 epidemic in Managua, Nicaragua. *Proc Natl Acad Sci U S A* 2018; **115**: 9294–9.

4 Gordon A, Kuan G, Mercado JC, *et al.* The Nicaraguan pediatric dengue cohort study: incidence of inapparent and symptomatic dengue virus infections, 2004-2010. *PLoS Negl Trop Dis* 2013; **7**: e2462.

5 Waggoner JJ, Gresh L, Mohamed-Hadley A, *et al.* Single-Reaction Multiplex Reverse Transcription PCR for Detection of Zika, Chikungunya, and Dengue Viruses. *Emerg Infect Dis* 2016; **22**: 1295–7.

6 Lanciotti RS, Calisher CH, Gubler DJ, Chang GJ, Vorndam AV. Rapid detection and typing of dengue viruses from clinical samples by using reverse transcriptase-polymerase chain reaction. *J Clin Microbiol* 1992; **30**: 545–51.

7 Balmaseda A, Sandoval E, Pérez L, Gutiérrez CM, Harris E. Application of molecular typing techniques in the 1998 dengue epidemic in Nicaragua. *Am J Trop Med Hyg* 1999; **61**: 893–7.

8 Waggoner JJ, Abeynayake J, Sahoo MK, *et al.* Single-reaction, multiplex, real-time rt-PCR for the detection, quantitation, and serotyping of dengue viruses. *PLoS Negl Trop Dis* 2013; **7**: e2116.

9 Balmaseda A, Zambrana JV, Collado D, *et al.* Comparison of Four Serological Methods and Two Reverse Transcription-PCR Assays for Diagnosis and Surveillance of Zika Virus Infection. *J Clin Microbiol* 2018; **56**. DOI:10.1128/JCM.01785-17.

10 Balmaseda A, Guzmán MG, Hammond S, *et al.* Diagnosis of dengue virus infection by detection of specific immunoglobulin M (IgM) and IgA antibodies in serum and saliva. *Clin Diagn Lab Immunol* 2003; **10**: 317–22.

11 Gordon A, Gresh L, Ojeda S, *et al.* Prior dengue virus infection and risk of Zika: A pediatric cohort in Nicaragua. *PLoS Med* 2019; **16**: e1002726.

12 Katzelnick LC, Gresh L, Halloran ME, *et al.* Antibody-dependent enhancement of severe dengue disease in humans. *Science* 2017; **358**: 929–32.

13 World Health Organization. Dengue guidelines for diagnosis, treatment, prevention and control : new edition. World Health Organization, 2009 https://iris.who.int/handle/10665/44188 (accessed March 2, 2024).

14 Bos S, Graber AL, Cardona-Ospina JA, *et al.* Protection against symptomatic dengue infection by neutralizing antibodies varies by infection history and infecting serotype. *Nat Commun* 2024; **15**: 382.

15 Fernández RJ, Vázquez S. Serological diagnosis of dengue by an ELISA inhibition method (EIM). *Mem Inst Oswaldo Cruz* 1990; **85**: 347–51.

16 Reed LJ, Muench H. A SIMPLE METHOD OF ESTIMATING FIFTY PER CENT ENDPOINTS. *Am J Epidemiol* 1938; **27**: 493–7.

17 Thongsripong P, Edgerton SV, Bos S, *et al.* Phylodynamics of dengue virus 2 in Nicaragua leading up to the 2019 epidemic reveals a role for lineage turnover. *BMC Ecol Evol* 2023; **23**: 58.
